# Evolution of a mating preference for a dual‐utility trait used in intrasexual competition in genetically monogamous populations

**DOI:** 10.1002/ece3.3145

**Published:** 2017-09-02

**Authors:** Caitlin A. Stern, Maria R. Servedio

**Affiliations:** ^1^ Department of Biology CB 3280 Coker Hall University of North Carolina Chapel Hill NC USA; ^2^ Present address: Caitlin A. Stern, Santa Fe Institute Santa Fe NM USA; ^3^ Present address: Interacting Minds Centre Aarhus University Aarhus C Denmark

**Keywords:** armament–ornament hypothesis, dual‐utility trait, intrasexual competition, mate choice, mating preference, monogamy

## Abstract

The selection pressures by which mating preferences for ornamental traits can evolve in genetically monogamous mating systems remain understudied. Empirical evidence from several taxa supports the prevalence of dual‐utility traits, defined as traits used both as armaments in intersexual selection and ornaments in intrasexual selection, as well as the importance of intrasexual resource competition for the evolution of female ornamentation. Here, we study whether mating preferences for traits used in intrasexual resource competition can evolve under genetic monogamy. We find that a mating preference for a competitive trait can evolve and affect the evolution of the trait. The preference is more likely to persist when the fecundity benefit for mates of successful competitors is large and the aversion to unornamented potential mates is strong. The preference can persist for long periods or potentially permanently even when it incurs slight costs. Our results suggest that, when females use ornaments as signals in intrasexual resource competition, males can evolve mating preferences for those ornaments, illuminating both the evolution of female ornamentation and the evolution of male preferences for female ornaments in monogamous species.

## 1 INTRODUCTION

The evolution of preferences for ornamental traits in genetically monogamous mating systems poses a puzzle for evolutionary biologists. How can preferences evolve when sexual selection is limited by the restriction that each individual can have only one mate (Andersson, 1986; Kirkpatrick, Price, & Arnold, 1990; O'Donald, 1980)? A further challenge is that mutual ornamentation and mutual mate choice are common in monogamous species (Dale, Dey, Delhey, Kempenaers, & Valcu, 2015; Kraaijeveld, Kraaijeveldsmit, & Komdeur, 2007; Tobias, Gamarra‐Toledo, García‐Olaechea, Pulgarín, & Seddon, 2011), and thus explanations for the evolution of both female and male preferences for ornamental traits are required. An emerging body of evidence suggests that ornaments are used in intrasexual resource competition by females (Brunton, Roper, & Harmer, 2016; Cain, Cockburn, & Langmore, 2015; Kraaijeveld, Gregurke, Hall, Komdeur, & Mulder, 2004; Krieg & Getty, 2016; Murphy, Hernandez‐Mucino, Osorio‐Beristain, Montgomerie, & Omland, 2009a; Murphy, Rosenthal, Montgomerie, & Tarvin, 2009b) as well as males (Chaine & Lyon, 2008; Evans & Hatchwell, 1992; Laubach, Blumstein, Romero, Sampson, & Foufopoulos, 2013; Part & Qvarnstrom, 1997; Pryke & Andersson, 2003; Pryke, Lawes, & Andersson, 2001), and these ornaments may also be preferred in mate choice (Mateos, 1998; Tarof, Dunn, & Whittingham, 2005; Griggio, Serra, Licheri, Monti, & Pilastro, 2006; Hoi & Griggio, 2008; Small, Cotton, Fowler, & Pomiankowski, 2009; reviewed by Berglund, Bisazza, & Pilastro, 1996; Hunt, Breuker, Sadowski, & Moore, 2009). These findings are consistent with the “armament–ornament” hypothesis, whereby a trait used in intrasexual competition becomes the object of a mating preference due to its pre‐existing association with qualities desirable in a mate (Berglund et al., 1996; Wiley & Poston, 1996). Here, we ask whether the armament–ornament process can operate in monogamous systems, leading to a preference for an ornamental trait used by the opposite sex in intrasexual resource competition. We employ a model that is equally applicable to the evolution of male or female preference, but this process is of especial interest in the case of male preferences for female ornaments, as we argue below.

In genetically monogamous species, mating preferences are expected to favor partners associated with higher fecundity matings because mating fecundity strongly influences reproductive success when each individual has only one mate (Andersson, 1994; Monaghan, Metcalfe, Houston, Monaghan, & Houston, 1996). Previous models of the evolution of a mating preference for an ornamental trait in genetically monogamous systems confirm the importance of an improvement in the fecundity or viability of preferred matings over unpreferred matings for preference evolution. Increases in fecundity and viability differ mechanistically: higher fecundity means that a greater number of offspring are produced relative to other matings, whereas higher viability means that a greater proportion of offspring survive relative to other matings. They can also differ in whether they exert direct or indirect selective forces on loci involved in mate choice. Fecundity selection acts on a mated pair and can thus cause direct selection on mating preferences, even if higher fecundity is a function of bearing the preferred trait. Viability selection, on the other hand, acts only indirectly on mate preferences, through the statistical associations between preferences and traits, if fitness is determined solely by the trait phenotype. In previous models, both factors ultimately have been found to facilitate selection for the coexistence of a mating preference and an ornamental trait. For example, in a one‐locus model of a male ornamental trait, O'Donald (1980) found that the ornament can be maintained when preferential matings have higher fecundity than random matings. In a three‐locus model asking whether viability differences can lead to evolution of a mating preference and ornamental trait in the absence of a Fisherian mating advantage (Fisher, 1930), Andersson (1986) assumed that all mated females have equal fecundity, but included a locus for phenotypic condition along with a female preference and a condition‐dependent male ornament. The model's results showed that the male trait and female preference could spread in a population because they become genetically associated with genes for high viability, due to the condition‐dependent nature of the trait (Andersson, 1986). Finally, a quantitative genetic model focused on breeding date as a means by which fecundity could become associated with preferential matings: incorporating a male trait, a female mating preference, and female breeding date, the model showed that costly male ornaments can evolve when early‐breeding females are more fecund and more likely to mate with preferred males (Kirkpatrick et al., 1990). Each of these models studied a female preference for a male ornament, leaving out male mating preferences. However, male mating preferences are also likely to be important in genetically monogamous systems. One might expect that, similar to female mating preferences in monogamy, male mating preferences might favor matings with higher fecundity or offspring viability. What processes could lead to a link between female fecundity or offspring viability and female ornamentation?

Intrasexual resource competition offers a mechanism by which a link between an ornamental trait and fecundity could emerge: if ornamented females, for example, are more likely to win competitions with other females for access to resources that enhance fecundity, such as high‐quality territories or food resources, males that prefer ornamented females will have more fecund mates (Tobias, Montgomerie, & Lyon, 2012). Emerging evidence supports the idea that, as suggested by multiple reviews covering the function and evolution of female ornamentation (Lyon & Montgomerie, 2012; Tobias et al., 2012; Webb et al., 2016; West‐Eberhard, 1983), female visual and vocal ornaments are often used in female–female competition over resources related to reproductive success (Brunton et al., 2016; Cain et al., 2015; Crowhurst, Zanollo, Griggio, Robertson, & Kleindorfer, 2012; Kraaijeveld et al., 2004; Krieg & Getty, 2016; Murphy et al., 2009a,b; Pryke, 2007; Stankowich & Caro, 2009; Watson & Simmons, 2010). For example, female black swans (*Cygnus atratus*) with more curled feathers are more likely to win female–female agonistic interactions, and number of curled feathers also predicts the ability to maintain territory ownership, which leads to higher offspring survivorship (Kraaijeveld et al., 2004). In New Zealand bellbirds (*Anthornis melanura*), females use song to defend breeding territories against other females, and female song rate predicts the number of young fledged (Brunton, Evans, Cope, & Ji, 2008; Brunton et al., 2016). Similar links among female song rate, female territorial defense against other females, and female offspring production occur in house wrens (*Troglodytes aedon*; Krieg & Getty, 2016) and superb fairy‐wrens (*Malurus cyaneus*; Cain et al., 2015). Correlations between ornamental traits and fecundity are also well known in males (Murphy, 2007; Palokangas et al., 1994; Preault, Chastel, Cezilly, & Faivre, 2005; Siefferman & Hill, 2003).

Do individuals prefer mates with ornaments that indicate success in intrasexual resource competition? The principle behind the armament–ornament hypothesis is that a preference for a trait possessed by successful competitors will more reliably lead to enhanced reproductive success than a preference for a trait unlinked to success in intrasexual competition (Berglund et al., 1996; Wiley & Poston, 1996). This is because the honesty of traits used in intrasexual competition over resources (including mates or food) is maintained through frequent contests (Berglund et al., 1996; Wiley & Poston, 1996). When these traits are subsequently used in mate choice, they thus become both armaments in the context of intrasexual selection and ornaments in the context of intersexual selection (Berglund et al., 1996). Female mating preferences for male ornaments used in male–male resource competition are well‐documented empirically, supporting the existence of these “dual‐utility” traits in multiple systems (Mateos, 1998; Tarof et al., 2005; Griggio et al., 2006; Hoi & Griggio, 2008; Small et al., 2009; reviewed by Berglund et al., 1996; Hunt et al., 2009). However, evidence that the armament–ornament process can shape male mating preferences for female traits is still slim (Tobias et al., 2012). Although several studies have demonstrated that females use ornamental traits in female–female resource competition (reviewed above), and other studies have documented male mating preferences for female ornamental traits (Amundsen, Forsgren, & Hansen, 1997; Cotton, Cotton, Small, & Pomiankowski, 2015; Tigreros, Mowery, & Lewis, 2014; Torres & Velando, 2005), few studies have tested whether males prefer the traits females use in intrasexual competition (Griggio, Valera, Casas, & Pilastro, 2005; Jones & Hunter, 1993, 1999; Murphy et al., 2009b; Pryke & Griffith, 2007). As interest in the armament–ornament hypothesis as a potential explanation for the evolution of male preferences for female ornaments grows, both theoretical evidence that this process can favor preference evolution and empirical evidence that it does so in nature are needed.

Here, we ask whether the armament–ornament process is a novel means by which a mating preference for an ornamental trait can evolve in monogamous mating systems. We explicitly consider the effect of an association between an ornamental trait and success in intrasexual resource competition (a dual‐utility trait) on the evolution of mating preferences under monogamy, an effect not examined by previous models. We assume that the ornamental trait is used in competition for resources, leading to greater resource acquisition and thus greater fecundity. To examine this effect alone, we exclude other processes known to facilitate preference evolution in monogamy, for example, that ornamental traits are indicators of good genes or are condition‐dependent (Andersson, 1986), or that mating according to a preference increases fecundity regardless of the mate's trait (O'Donald, 1980). We allow both sexes to express the ornamental trait because sexually monomorphic ornamentation is common in monogamous species, and expression in both sexes is also thought to be common for dual‐utility traits (Dale et al., 2015; Kraaijeveld et al., 2007; Tobias et al., 2011). Because we assume strict monogamy with an equal sex ratio, individuals do not differ in mating success. Individuals of the choosing sex benefit from mating with mates that hold more resources, but do not vary intrinsically in their fecundity (c.f. Kirkpatrick et al., 1990). This model thus functions as a proof‐of‐concept test of the hypothesis that the armament–ornament process is a potential route by which a male preference for a female trait used in intrasexual resource competition can evolve, testing the logic of this verbal explanation in a way analogous to using empirical data to test hypotheses (Servedio et al., 2014).

We find that a mating preference for a dual‐utility trait associated with success in intrasexual resource competition can increase in frequency in a monogamous population, supporting the idea that the armament–ornament process provides a novel path by which preferences for ornamental traits can evolve even under strict genetic monogamy. This pattern persists in the face of a weak cost to the mating preference, and applies equally to male and female preferences.

## 2 THE MODEL

We constructed a population genetic model to study the evolution of a mating preference for a trait used in intrasexual resource competition in genetically monogamous mating systems. Our model includes two haploid loci: the locus T controls an ornamental trait that is used in intrasexual resource competition, whereas the locus P controls a mating preference. The model applies equally to male preferences for female traits and female preferences for male traits; we present the model in terms of the evolution of male preferences in order to emphasize its application to understanding male mate choice and female ornamentation in monogamous systems.

The trait and preference loci each have two alleles. Females carrying the T_2_ allele express the ornamental trait, whereas T_1_ females are unornamented. Males carrying the P_2_ allele express an aversion to unornamented females, whereas P_1_ males do not express a mating aversion. An aversion to T_1_ females is effectively a preference for T_2_ females, and simplifies the calculations. Frequencies of the alleles are denoted by lower case, for example, $p_{2}$. The genotypes T_1_P_1_, T_1_P_2_, T_2_P_1_, and T_2_P_2_ occur with frequencies $x_{1}$, $x_{2}$, $x_{3}$, and $x_{4}$, respectively. The life cycle consists of mutation, viability selection, mate choice, fecundity selection, and recombination. In each generation, T_2_ mutates to T_1_ at rate μ. Biased mutation against the ornamental trait is expected when the trait is complex and there are many mutations that can degrade it (e.g., Pomiankowski, Iwasa, & Nee, 1991). The genotype frequencies after mutation are $x_{1\mu }=x_{1}+\mu x_{3}$, $x_{2\mu }=x_{2}+\mu x_{4}$, $x_{3\mu }=x_{3}\left(1-\mu \right)$, and $x_{4\mu }=x_{4}\left(1-\mu \right)$.

The ornamental trait T_2_ carries a viability cost s (0≤s<1 ) and is expressed in both sexes. We assume that the ornament is expressed in both sexes because sexually monomorphic ornamentation is common in monogamous species (Dale et al., 2015; Kraaijeveld, 2014; Kraaijeveld et al., 2007; Tobias et al., 2011), which means that this assumption brings the model into accordance with the biology of the species of interest. Sexually monomorphic ornamentation is also required for our assumption that the frequencies of males and females in the population are equal, which significantly simplifies our analyses. Males with the preference allele P_2_ also suffer a fixed cost c (0≤c<1 ). The genotype frequencies for males after viability selection are thus given by:

(1) $${x_{mi}}^{\prime }=\frac{\left(1-ys\right)\left(1-lc\right)x_{i\mu }}{1-cx_{2\mu }-sx_{3\mu }-\left(s+c-sc\right)x_{4\mu }}$$

where y=0 when i=1 or i=2 and y=1 when i=3 or i=4, and l=0 when i=1 or i=3, and l=1 when i=2 or i=4. We consider only very weak preference costs that do not substantially alter the sex ratio. The genotype frequencies for females after viability selection are given by:

(2) $${x_{fj}}^{\prime }=\frac{\left(1-ys\right)x_{j\mu }}{\sum_{i}\left(1-ys\right)x_{j\mu }}$$

where y=0 when j=1 or j=2 and y=1 when j=3 or j=4.

During mating, males carrying allele P_2_ express an aversion to T_1_ females of strength ρ, where 0≤ρ≤1 . This aversion is a population‐based measure: ρ denotes the reduction in the frequency of matings between P_2_ males and T_1_ females compared to random pairing. A population‐level measure of aversion strength is used here because, due to the sampling without replacement required to capture a monogamous mating system, per‐encounter measures are not stable over time: sampling without replacement means that the frequencies of male and female types in the population change continuously throughout the pairing process. The frequency of matings between a P_*a*_ male and a T_*b*_ female is denoted by $q_{ab}$. The frequency of matings between P_2_ males and T_1_ females, which are unpreferred, would thus be $q_{21}=p_{2}^{\prime }{t_{1}}^{\prime }\left(1-\rho \right)$, where ${p_{a}}^{\prime }$ is the frequency of P_*a*_ among males after viability selection, and ${t_{b}}^{\prime }$ is the frequency of T_*b*_ among females after viability selection. We calculate the remaining mating frequencies under the restriction that, under monogamy, mates are sampled without replacement, which means that females chosen as social mates are no longer part of the mating pool (we assume an equal sex ratio). We further assume that no male or female goes unmated. Thus, the T_1_ females not mated by P_2_ males must be mated by P_1_ males; the frequency of matings between P_1_ males and T_1_ females is therefore $q_{11}={t_{1}}^{\prime }-q_{21}$. Similarly, all P_2_ males that do not mate with T_1_ females logically must mate with T_2_ females; thus, the frequency of matings between P_2_ males and T_2_ females is $q_{22}={p_{2}}^{\prime }-q_{21}$. Finally, because all P_1_ males that do not mate with T_1_ females must mate with T_2_ females, the frequency of matings between P_1_ males and T_2_ females is $q_{12}={p_{1}}^{\prime }-q_{11}$. The net result of these assumptions is that males that cannot mate with the type of female that they prefer are assumed to mate with another female. Note that our approach limits the generality of the model: we can only consider cases in which the frequency of the preference is lower than the frequency of the trait, which encompasses the initial evolution of a rare preference. We were unable to find a more general formulation of the model that also retained both sampling without replacement and equal mating success between males and females. However, the restriction to studying cases in which preference frequency is lower than trait frequency is unlikely to limit our understanding of the evolution of trait and preference (see the logic presented in the Results and Discussion). The frequency of matings between each male genotype $x_{mi}$ and each female genotype $x_{fj}$ is

(3) $$m_{ij}=\left(\frac{{x_{mi}}^{\prime }}{{p_{a}}^{\prime }}\right)\left(\frac{{x_{fj}}^{\prime }}{{t_{b}}^{\prime }}\right)q_{ab}.$$

All males have equal mating success, as do all females, conforming to strict monogamy. Although strictly equal mating success for all individuals in a population is likely rare in nature, excluding sexual selection allows us to study the evolution of a mating preference for a dual‐utility trait under monogamy without confounding factors.

Next, fecundity selection occurs. The female trait is used in female–female resource competition, such that females possessing the trait gain relatively more resources than females without the trait: resource gains during competition are relative, with gains by females with the trait and gains by females without the trait accruing in the ratio 1 +  *f*:1. This relative advantage in resource gains by T_2_ females holds constant as long as both T_1_ and T_2_ females are present. Thus, no modifications to this approach are necessary to incorporate density‐dependence of resource gains. Note that we have not explicitly incorporated the dynamics of competition among females, which is necessary for complete understanding of ornamental trait evolution. Because our focus here is on preference evolution, we simplify the analyses by treating the ornamental trait as though it indicates a greater ability to access resources, which are not limiting.

Any male, regardless of his genotype, that mates with a T_2_ female receives the fecundity benefit f, where 0≤f . Normalized by mean fecundity, the fecundity of a mating between a male of genotype $x_{mi}$ and a female of genotype $x_{fj}$ is

(4) $$h_{ij}=\frac{\left(1+gf\right)m_{ij}}{\sum_{i}\sum_{j}\left(1+gf\right)m_{ij}}$$

where g=0 if j=1 or 2 and g=1 if j=3 or 4.

Free recombination occurs between the two loci and is followed by zygote production. We develop recursion equations in terms of the genotype frequencies, and transform them to calculate allele frequencies and the linkage disequilibrium (D) between loci P and T, using *Mathematica* (Wolfram Research Inc, 2010). The recursion equations Δ p_2_ and Δ t_2_ are presented in the Appendix S1. The *Mathematica* file showing the model derivation and our analyses is included as supporting information, and is available from the Dryad Digital Repository (https://doi.org/10.5061/dryad.s0vc7).

### 2.1 Simulations

We used numerical iteration of the recursion equations to examine the changes in allele frequencies with different parameter values, truncating results when allele frequencies violated the model requirement that the frequency of the preference is lower than the frequency of the trait ($p_{2}<t_{2}$; see below for discussion of generalizing past this truncation). We sought to identify the regions in which a preference for a trait used in intrasexual resource competition could increase and/or persist when the trait was already relatively common (as might be the case if the trait indicates a superior competitive ability). Our starting conditions for all displayed results are $t_{2}=0.8$ and $p_{2}=0.05$; we ran additional simulations with a variety of other starting conditions (including low $t_{2}$) to verify that these starting conditions yielded typical results (see Appendix S1). For comparison, results for the starting conditions $t_{2}=0.1$ and $p_{2}=0.05$ are shown in the supporting information (Figs. S1 and S2).

## 3 RESULTS

We first examine the dynamics of the model when preference costs are absent, in order to isolate the effects of the parameters on changes in the frequencies of the trait and preference alleles. We follow this with simulations that indicate evolutionary outcomes over longer time scales. Finally, we discuss the effects on the results of the introduction of preference costs.

### 3.1 Allele frequency change in a single generation

Examination of the preference and trait frequencies after one time step, $p_{2,t_{1}}$ and $t_{2,t_{1}}$, considered in this case when c=0, allows us to study the effects of the parameters on the frequencies of the preference and trait alleles. Because $p_{2,t_{1}}$ and $t_{2,t_{1}}$ capture only the changes in preference and trait allele frequencies over a single generation, these analyses do not reveal long‐term evolutionary trajectories. However, studying change in a single generation allows us to use a general, analytical approach, helping us to understand how the parameters influence the preference and trait allele frequencies. In the next section, we look at evolution over the longer term using simulations. Full expressions for $p_{2,t_{1}}$ and $t_{2,t_{1}}$, as well as the details of all analyses described here, are shown in the supporting information.

Preference frequency always increases ($\partial p_{2,t_{1}}/\partial \rho >0$) as aversion strength (ρ) increases. The increase in the preference frequency with ρ occurs because a stronger aversion facilitates greater linkage disequilibrium between preference and trait. Unless s>0.5, which seems unrealistically high, it is only possible for the frequency of the preference allele to increase ($\partial p_{2,t_{1}}/\partial f>0$) as the fecundity benefit (f) increases when $\rho <\frac{1-s}{s\left(t_{2}\mu -t2+1\right)}$, a condition that holds for a range of realistic parameter values. The increase in $p_{2}$ with the fecundity benefit is due to direct selection (the larger the fecundity benefit conferred by mating with a T_2_ female, the larger is the advantage of having a preference for, and hence being differentially paired with, T_2_ females), but is also expected to have a contribution from indirect selection: the direct selection fecundity benefit to T_2_ females would lead to an increase in P_2_ via linkage disequilibrium. Finally, analyzing the effect of the viability cost on preference frequency shows that preference frequency is more likely to decrease ($\partial p_{2,t_{1}}/\partial s<0$) as the viability cost (s) increases when linkage disequilibrium is large. This result is expected because selection lowers the trait frequency and linkage disequilibrium mediates the effect of this cost on the preference.

We further investigate the importance of ρ for preference evolution by studying the case when ρ=0, finding that, in this case, preference frequency will only increase as the fecundity benefit increases when linkage disequilibrium is greater than zero. This result demonstrates that ρ is crucial for the evolution of the preference, because only when ρ>0 can the preference increase in the population without pre‐existing linkage disequilibrium between the preference and the trait. Furthermore, linkage disequilibrium can be shown not to build up when ρ=0.

The frequency of the trait allele increases as the fecundity benefit ($\partial t_{2,t_{1}}/\partial f>0$) and the aversion strength ($\partial t_{2,t_{1}}/\partial \rho >0$) increase, but decreases as the viability cost ($\partial t_{2,t_{1}}/\partial s<0$) increases for all realistic parameter values. The increase in $t_{2}$ with the fecundity benefit is likely primarily due to direct selection: females with the trait allele have higher relative fitness when the fecundity benefit is higher. The increase in $t_{2}$ with aversion strength is instead due to the role of the aversion in facilitating the build‐up of linkage disequilibrium between the preference and trait loci, allowing changes in the preference frequency from selective forces such as fecundity selection to lead to changes in the trait frequency due to indirect selection. The decrease in $t_{2}$ with increasing viability cost is clearly due to the direct effect of lower survivorship.

Note that, when s=0 and f=0, we find no dependence of $t_{2}\;$and $p_{2}$ on ρ, indicating that there is no change in the trait or preference allele frequencies from aversion (ρ) alone. This verifies that indeed no sexual selection occurs in this model of monogamy.

### 3.2 Simulation results

**Figure 1:**
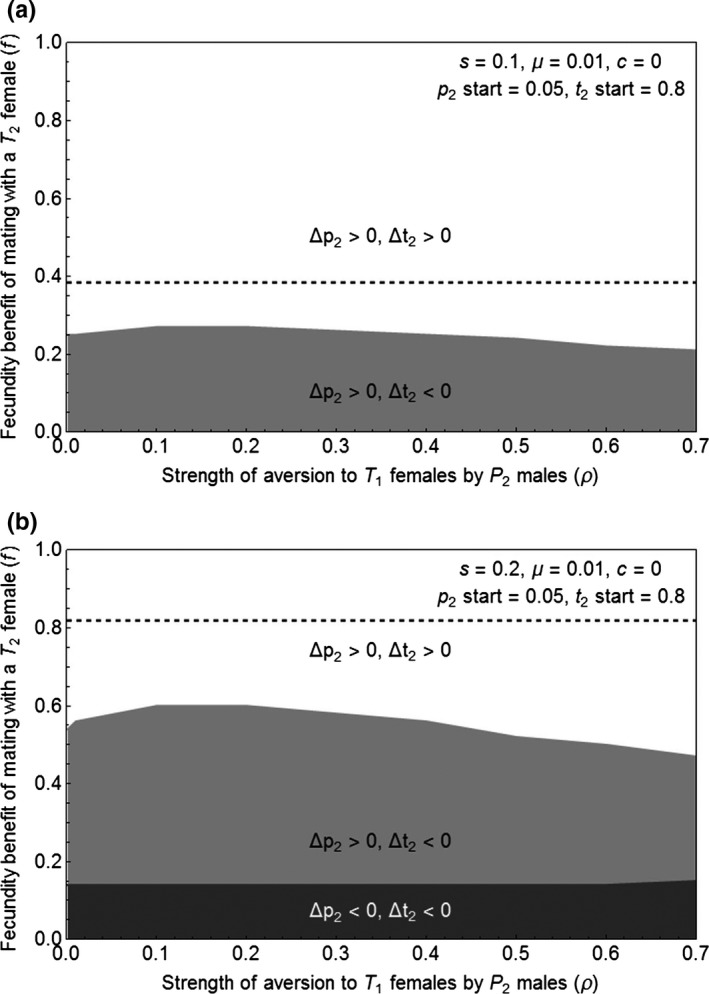
(a) The region in which the frequencies of the preference and the ornamental trait both increase (white) when there is no cost of the preference (c=0) and biased mutation occurs at a low level against the trait (μ=0.01), mimicking the case in which the trait is complex and there are many mutations that can degrade it. (b) With a higher viability cost to carrying the trait allele (s=0.2 rather than s=0.1 in panel a), the region in which the frequencies of both the preference and the ornamental trait increase is smaller. In both panels, the dashed line indicates the threshold value of f above which the trait frequency increases in the absence of the preference allele ($p_{2}=0$). Note that the lowest value of ρ in the simulations is 0.001 because, when ρ=0, $p_{2}$ cannot increase with f unless we assume a starting level of linkage disequilibrium greater than 0 (see supporting information for analyses)

**Figure 2:**
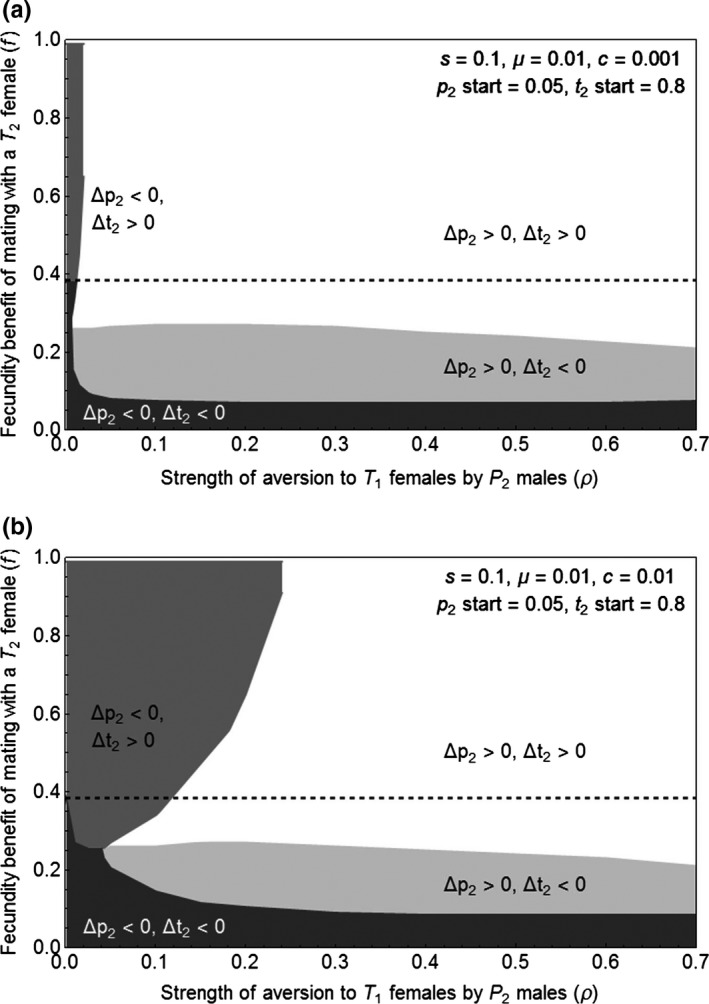
A cost of the preference reduces the size of the region in which the preference and trait allele increase, but this region (shown in white) still occurs. Displayed are results for cost of preference c=0.001 (panel a), and cost of preference c=0.01 (panel b). As in Figure 1, in both panels, the dashed line indicates the threshold value of f above which the trait frequency increases in the absence of the preference allele, and ρ=0.001 is the lowest value of ρ in the simulations

We find that the trait and preference alleles can both increase in frequency, including when there is a cost to the preference, although the size of the region in which Δp_2_ > 0 and Δt_2_ > 0 decreases with the magnitude of the cost. We compare these results to the region in which Δt_2_ > 0 in the absence of the preference. We can determine when $t_{2}$ will persist in the absence of the preference by evaluating Δt_2_ with $p_{2}=0$, which shows that Δt_2_ depends upon $t_{2}$, f, s, and μ (shown in supporting information). When $p_{2}=0$, c does not appear in Δt_2_, which means that the results apply whether or not a preference cost is present; the aversion strength ρ is also absent. When we employ the parameter values used in our displayed simulations, s=0.1 or s=0.2 and μ=0.01, whether Δt_2_ is positive depends upon f and the starting value of $t_{2}$. Using starting $t_{2}=0.8$, we find that Δ $t_{2}$ is expected to persist in the absence of the preference, when f>0.38 for s=0.1 and when f>0.82 for s=0.2. These thresholds for the persistence of the ornamental trait in the absence of the preference are displayed as dashed lines on Figures 1a, b and 2a, b.

### 3.3 No cost of preference

When the preference confers no cost, the trait and preference allele frequencies increase over a large region (Figure 1a); the size of this region is smaller when the cost of carrying the trait allele is higher (Figure 1b), but the trait and preference allele frequencies still increase for a large range of combinations of fecundity benefit (f) and aversion strength (ρ) values. The presence of the preference contributes to the increase in the trait (compare the white regions to the dashed lines in Figure 1).

In all simulations with no cost of preference, $p_{2}$ increases more rapidly than, and eventually exceeds, $t_{2}$. Although our model does not allow us to examine the long‐term dynamics of allele frequency change when $p_{2}$ evolves to exceed $t_{2}$, we expect that the regions in which both $t_{2}$ and $p_{2}$ are increasing represent a set of cases in which a dual‐utility trait and corresponding preference could be maintained: we see no logical mechanism whereby, if both trait and preference frequencies are increasing, the trait frequency could begin to decrease after being surpassed by the preference frequency (see Discussion), and without a cost of preference we never see the preference decreasing when the trait is not lost. We expect that a dual‐utility trait cannot be maintained in the grey regions where $t_{2}$ is decreasing before the point at which $p_{2}$ exceeds $t_{2}$. If the trait is lost, the preference will then be evolutionarily neutral, and remain at whatever frequency it has attained.

### 3.4 Cost of preference

When the preference confers a cost, the trait and preference allele frequencies still increase over a significant region of parameter space; this region is larger when the cost of preference is very low (c=0.001; Figure 2a) than when it is higher (c=0.01; Figure 2b).

The addition of a higher cost of the male preference also results in the appearance of a region of parameter space in which Δp_2_ is negative and Δt_2_ is positive, and $p_{2}$ does not evolve to exceed $t_{2}$ (Figure 2b). In this region, $t_{2}$ always increases to a high constant frequency in our simulations, whereas $p_{2}$ gradually declines. After allele T_2_ has reached a high frequency in the population, the cost of the preference is the only significant force left on allele P_2_ in the model, causing the decline in $p_{2}$. However, because this decline takes place over hundreds or thousands of generations with a weak cost, the preference might still be observed in nature.

In the other regions where $p_{2}$ increases, it always eventually exceeds $t_{2}$, which means again that we cannot follow the long‐term dynamics of the trait and preference alleles. In areas in which both $t_{2}$ and $p_{2}$ are increasing, it is likely that $p_{2}$ will start to drop in frequency when $t_{2}$ becomes too high, since the benefit of the costly preference allele would be lost if there were very little trait variation (mutation prevents trait fixation). If the trait continues to increase to a very high frequency despite a lowering of the preference frequency, as would be expected when there is a high fecundity benefit (above the dashed lines in the figures), it is likely that the preference would be lost altogether.

If, however, the trait begins to drops in frequency when the preference becomes too low (as is expected below the dashed lines, where the preference is needed for the trait to increase), then this increasing trait variation may allow the preference to increase again. We note that with weak costs $p_{2}$ drops very slowly, and that in spot checks of the white region below the dashed line $p_{2}$ increases even when $t_{2}$ is at a very low frequency ($t_{2}=0.1$), so it is unlikely that $t_{2}$ will be lost before the preference again begins to spread, and we anticipate that both $t_{2}$ and $p_{2}$ may be maintained in this region.

## 4 DISCUSSION

We find that a preference for an ornamental trait used in intrasexual competition can indeed evolve, at least to the point of being present for long periods of time, and affect the evolution of the ornament, when mating occurs only within monogamous pairs. This mechanism can thus provide an explanation for the existence of male preferences for female ornaments that are indicators of competitive success. In our model, the fecundity benefit derived from success in intrasexual competition for resources provides direct and indirect selection on both traits and preferences. Direct selection acts on both trait and preference through the fecundity benefit: the larger the fecundity benefit, the larger the advantage enjoyed by successful competitors and the males that prefer them. Because the preference and trait are in linkage disequilibrium, the direct benefits on each locus also confer a benefit by indirect selection. Unlike fecundity selection, the aversion parameter does not apply selection on either of the loci. Because males and females have equal mating success in this monogamous system, aversion does not result in sexual selection. It does, however, result in the accumulation of linkage disequilibrium between the trait and preference loci, which allows for and strengthens the indirect selection effects described above.

Introducing a cost of the mating preference leads to a reduction in the size of the region in which both the preference and trait frequencies increases. However, the preference and trait allele frequencies still increase across a significant region of parameter space, indicating that moderate fecundity benefit and aversion strength values may be sufficient to allow the initial evolution of a costly preference for an ornamental trait. Our current model requires that the frequencies of males and females in the population are equal and thus precludes large costs of the preference allele; however, an expansion of this model that includes mutual mate choice could circumvent this restriction.

Our results demonstrate that the process proposed in the armament–ornament model is a means by which a preference for an ornamental trait can evolve under monogamy. Although our model is restricted to studying cases in which the frequency of the preference is lower than the frequency of the trait, we see no reason to suspect that, in the case without preference costs, when $p_{2}$ surpasses $t_{2}$ the forces should shift such that $t_{2}$ would decrease if both were initially increasing (recall that aversion places no direct selection on the trait, and the preference and trait are positively correlated). Thus, in this case, both the preference and trait would be expected to fix or remain at a high mutation‐selection balance. Furthermore, even if $p_{2}$ began to eventually decrease, as may occur when the preference is costly and trait frequencies are very high (see above), once $p_{2}$ was again lower than $t_{2}$, the dynamics seem likely to return to those captured in our model: $p_{2}$ could thus once again increase (or an equilibrium could be reached; see arguments in Results section). Therefore, we expect that, even with preference costs, P_2_ is likely to be maintained in many cases in which traits and preferences initially increase.

To our knowledge, the evolution of mating preferences for traits that predict success in intrasexual resource competition has not previously been studied from a theoretical perspective. We incorporated intrasexual competition into our model by assuming that individuals bearing the trait were successful in competing for resources that enhance a mate's fecundity, whereas individuals without the trait were not. Empirical studies have provided evidence that increased ornamentation is associated with improved resources defense in both males (Chaine & Lyon, 2008; Evans & Hatchwell, 1992; Laubach et al., 2013; Part & Qvarnstrom, 1997; Pryke & Andersson, 2003; Pryke et al., 2001) and females (Brunton et al., 2016; Cain et al., 2015; Kraaijeveld et al., 2004; Krieg & Getty, 2016). The implications of our results for male mating preferences and female ornamental traits are particularly interesting in the light of recent suggestions that female ornaments may be more frequently employed in resource competition than are male ornaments (Dale et al., 2015; Tobias et al., 2012; Webb et al., 2016). Empirically, our results suggest the need for well‐supported examples of male mating preferences for female ornaments that are used in intrasexual resource competition.

Recent interest in the conditions under which male mating preferences are expected to evolve has largely focused on polygynous populations (Servedio & Lande, 2006; Servedio, 2007; Nakahashi, 2008; South, Arnqvist, & Servedio, 2012; but see Ihara & Aoki, 1999; Kokko & Johnstone, 2002). Our result that a male mating preference for an ornamental female trait can persist in a monogamous population thus provides an interesting comparison to results from models of polygyny. Because all males acquire mates in monogamy as we have modeled it here, the direct selection against male mate choice that emerges from competition for mates in polygynous systems (Servedio & Lande, 2006) is removed. In addition, the evolution of a male preference is facilitated by the fact that, in the present model, males with a preference are more likely to mate with females that are successful in intrasexual competition for resources and thus have higher fecundity matings. Male mate choice in polygyny is not expected to evolve when female ornamental traits are arbitrary, but can likewise evolve when those traits are associated with higher fecundity (Servedio, 2007; Servedio & Lande, 2006).

In previous models, the evolution of preferences for ornamental traits occurred in genetically monogamous mating systems when the process was given a “boost” by factors including a fecundity benefit of preferential matings, condition‐dependence of the trait, and increased representation of ornamented males among the mates of higher‐fecundity females (Andersson, 1986; Kirkpatrick et al., 1990; O'Donald, 1980). The finding that an armament–ornament process similarly provides a boost that can lead to the evolution, and likely the persistence, of a preference for an ornamental trait in genetic monogamy expands the known set of explanations for this phenomenon.
