# Supplementary material for: Evolution of a mating preference for a dual‐utility trait used in intrasexual competition in genetically monogamous populations
Source: Ecol Evol. 2017 Sep 2;7(19):8008–16. doi: 10.1002/ece3.3145 (PMC5632625; doi:10.1002/ece3.3145)
Supplement: Supplementary file 1 [file ECE3-7-8008-s001.docx]

**APPENDIX**

**1. Recursions for** $\boldsymbol{\Delta}\boldsymbol{t}_{\boldsymbol{2}}$ **and** $\boldsymbol{\Delta}\boldsymbol{p}_{\boldsymbol{2}}$

The recursion equations for $t_{2}$ and $p_{2}$ are as follows (see the *Mathematica* (Wolfram Research Inc. 2010) files on Dryad for the full derivation):

**,**

and

where

The parameter $\alpha$ represents viability selection against T_2_ in males; as noted in the main text, we set $\alpha=s$ for all analyses to justify an equal sex ratio. Parameter $c$ is left at an arbitrary value in these equations. It can be seen that there are components of both recursions that do not depend on the linkage disequilibrium, $D$, indicating that both alleles evolve partly by direct selection.

**2. Parameter and starting values tested**

In the simulations, we studied the parameter ranges $0.01\leq f\leq0.99$ and $0.001\leq\rho\leq0.7$ in increments ranging from 0.001 to 0.1. We studied three possible preference costs ($c=0$, $c=0.001$, and $c=0.01$) and two possible viability costs ($s=0.1$ and $s=0.2$). We used the mutation rate $\mu=0.01$. We examined starting conditions including $t_{2}=0.8, p_{2}=0.1$, and $p_{2}=0.05$ with $t_{2}$ ranging from $0.1$ to $0.8$ in increments of $0.1$.
