# Supplementary material for: Evolution of a mating preference for a dual‐utility trait used in intrasexual competition in genetically monogamous populations
Source: Ecol Evol. 2017 Sep 2;7(19):8008–16. doi: 10.1002/ece3.3145 (PMC5632625; doi:10.1002/ece3.3145)
Supplement: Supplementary file 2 [file ECE3-7-8008-s002.pdf]

Supplementary Material for “Evolution of a male mating preference for a dual-utility trait used in female intrasexual competition in genetically monogamous populations”

Contents:

1. Analysis of the model and generation of the main text figures

A. Derivation of the model

B. Analysis of the model

C. Simulations

D. Figures

2. Supplementary figures

1. Analysis of the model and generation of the main text figures

A. Derivation of the model

Allele frequencies and variables

x1 = freq of T1P1 genotype

Printed by Wolfram Mathematica Student Edition

x2 = freq of T1P2 genotype

x3 = freq of T2P1 genotype

x4 = freq of T2P2 genotype

s = viability cost of carrying the trait allele for females

α = viability cost of carrying the trait allele for males

μ = rate at which T2 mutates to T1 in each generation

c = cost of the preference allele

ρ = strength of aversion that P2 males express to T1 females

f = fecundity benefit gained by males that mate with a T2 female (all matings with T2 females have fecundity = 1+f, where 0 <= f, while matings with T1 females have fecundity = 1)

```
In[1]:= ClearAll[x1, x2, x3, x4, x1vf, x2vf, x3vf, x4vf, x1vm,
         x2vm, x3vm, x4vm, ρ, s, r, f, μ, F, p2, t2, diseq, α, c]

In[2]:= α = s;
```

If analyzing with no cost of preference, set c = 0 here:

```
In[3]:= c = 0;
```

Mutation and viability selection

xiμ = frequency of xi individuals after mutation

In[4]:=
```
x1μ = x1 + μ * x3
x2μ = x2 + μ * x4
x3μ = x3 * (1 - μ)
x4μ = x4 * (1 - μ)
```

Out[4]= $x1 + x3\,\mu$

Out[5]= $x2 + x4\,\mu$

Out[6]= $x3\,(1 - \mu)$

Out[7]= $x4\,(1 - \mu)$

viability selection:
xivf = frequency of xi males after viability selection
xivm = frequency of xi females after viability selection

```
In[8]:= x1vf =
   FullSimplify[x1μ / (x1μ + x2μ * (1 - c) + x3μ * (1 - α) + x4μ * (1 - α) * (1 - c))]
  x2vf = FullSimplify[(x2μ * (1 - c)) /
     (x1μ + x2μ * (1 - c) + x3μ * (1 - α) + x4μ * (1 - α) * (1 - c))]
  x3vf = FullSimplify[(x3μ * (1 - α)) /
     (x1μ + x2μ * (1 - c) + x3μ * (1 - α) + x4μ * (1 - α) * (1 - c))]
  x4vf = FullSimplify[(x4μ * (1 - α) * (1 - c)) /
     (x1μ + x2μ * (1 - c) + x3μ * (1 - α) + x4μ * (1 - α) * (1 - c))]
```

Out[8]= $$\frac{x1 + x3\,\mu}{x1 + x2 + (x3 + x4)\,(1 + s\,(-1 + \mu))}$$

Out[9]= $$\frac{x2 + x4\,\mu}{x1 + x2 + (x3 + x4)\,(1 + s\,(-1 + \mu))}$$

Out[10]= $$\frac{(-1 + s)\,x3\,(-1 + \mu)}{x1 + x2 + (x3 + x4)\,(1 + s\,(-1 + \mu))}$$

Out[11]= $$\frac{(-1 + s)\,x4\,(-1 + \mu)}{x1 + x2 + (x3 + x4)\,(1 + s\,(-1 + \mu))}$$

In[12]:=
```
x1vm = FullSimplify[x1μ / (x1μ + x2μ + x3μ * (1 - s) + x4μ * (1 - s))]
x2vm = FullSimplify[x2μ / (x1μ + x2μ + x3μ * (1 - s) + x4μ * (1 - s))]
x3vm = FullSimplify[(x3μ * (1 - s)) / (x1μ + x2μ + x3μ * (1 - s) + x4μ * (1 - s))]
x4vm = FullSimplify[(x4μ * (1 - s)) / (x1μ + x2μ + x3μ * (1 - s) + x4μ * (1 - s))]
```

Out[12]= $$\frac{x1 + x3\,\mu}{x1 + x2 + (x3 + x4)\,(1 + s\,(-1 + \mu))}$$

Out[13]= $$\frac{x2 + x4\,\mu}{x1 + x2 + (x3 + x4)\,(1 + s\,(-1 + \mu))}$$

Out[14]= $$\frac{(-1 + s)\,x3\,(-1 + \mu)}{x1 + x2 + (x3 + x4)\,(1 + s\,(-1 + \mu))}$$

Out[15]= $$\frac{(-1 + s)\,x4\,(-1 + \mu)}{x1 + x2 + (x3 + x4)\,(1 + s\,(-1 + \mu))}$$

Mating

PiTjs = frequency of matings between Pi males and Tj females

For P1 males, the availability of T2 females for mating is affected by the frequency of P2 males

In[16]:=
```
P2T1s = Simplify[((x2vf + x4vf) / (x1vf + x2vf + x3vf + x4vf)) *
    ((x1vm + x2vm) / (x1vm + x2vm + x3vm + x4vm)) * (1 - ρ)];
P1T1s = Simplify[((x1vf + x2vf) / (x1vf + x2vf + x3vf + x4vf)) - P2T1s];
P2T2s = Simplify[((x2vf + x4vf) / (x1vf + x2vf + x3vf + x4vf)) - P2T1s];
P1T2s = Simplify[((x1vf + x3vf) / (x1vf + x2vf + x3vf + x4vf)) - P1T1s];
```

Total frequency of P1 males: x1+x3

of P2 males: x2+x4

of T1 females: x1+x2

of T2 females: x3+x4

Within each mating type, the relative frequency of genotypes:
male P2T2: x4/(x2+x4)
male P2T1: x2/(x2+x4)
male P1T2: x3/(x1+x3)
male P1T1: x1/(x1+x3)
female P2T2: x4/(x3+x4)
female P1T2: x3/(x3+x4)
female P2T1: x2/(x1+x2)
female P1T1: x1/(x1+x2)

mij = frequency of xi male genotype mating with xj female genotype

Frequency of x4 male genotype mating with x3 female genotype = m43 = relative freq of the male genotype among P2 males * relative freq of the female genotype among T2 females * relative freq of a P2T2 mating

In[20]:=
```
m11 = FullSimplify[(x1vf / (x1vf + x3vf)) * (x1vm / (x1vm + x2vm)) * P1T1s];
m12 = FullSimplify[(x1vf / (x1vf + x3vf)) * (x2vm / (x1vm + x2vm)) * P1T1s];
m13 = FullSimplify[(x1vf / (x1vf + x3vf)) * (x3vm / (x3vm + x4vm)) * P1T2s];
m14 = FullSimplify[(x1vf / (x1vf + x3vf)) * (x4vm / (x3vm + x4vm)) * P1T2s];
m21 = FullSimplify[(x2vf / (x2vf + x4vf)) * (x1vm / (x1vm + x2vm)) * P2T1s];
m22 = FullSimplify[(x2vf / (x2vf + x4vf)) * (x2vm / (x1vm + x2vm)) * P2T1s];
m23 = FullSimplify[(x2vf / (x2vf + x4vf)) * (x3vm / (x3vm + x4vm)) * P2T2s];
m24 = FullSimplify[(x2vf / (x2vf + x4vf)) * (x4vm / (x3vm + x4vm)) * P2T2s];
m31 = FullSimplify[(x3vf / (x1vf + x3vf)) * (x1vm / (x1vm + x2vm)) * P1T1s];
m32 = FullSimplify[(x3vf / (x1vf + x3vf)) * (x2vm / (x1vm + x2vm)) * P1T1s];
m33 = FullSimplify[(x3vf / (x1vf + x3vf)) * (x3vm / (x3vm + x4vm)) * P1T2s];
m34 = FullSimplify[(x3vf / (x1vf + x3vf)) * (x4vm / (x3vm + x4vm)) * P1T2s];
m41 = FullSimplify[(x4vf / (x2vf + x4vf)) * (x1vm / (x1vm + x2vm)) * P2T1s];
m42 = FullSimplify[(x4vf / (x2vf + x4vf)) * (x2vm / (x1vm + x2vm)) * P2T1s];
m43 = FullSimplify[(x4vf / (x2vf + x4vf)) * (x3vm / (x3vm + x4vm)) * P2T2s];
m44 = FullSimplify[(x4vf / (x2vf + x4vf)) * (x4vm / (x3vm + x4vm)) * P2T2s];
```

Check: should add up to relative frequency of each male genotype, and total to 1:

In[36]:=
```
m1j = FullSimplify[m11 + m12 + m13 + m14];
m2j = FullSimplify[m21 + m22 + m23 + m24];
m3j = FullSimplify[m31 + m32 + m33 + m34];
m4j = FullSimplify[m41 + m42 + m43 + m44];
FullSimplify[m1j + m2j + m3j + m4j];
```

In[41]:=
```
mi1 = FullSimplify[m11 + m21 + m31 + m41];
mi2 = FullSimplify[m12 + m22 + m32 + m42];
mi3 = FullSimplify[m13 + m23 + m33 + m43];
mi4 = FullSimplify[m14 + m24 + m34 + m44];
FullSimplify[mi1 + mi2 + mi3 + mi4];
```

Printed by Wolfram Mathematica Student Edition

Fecundity selection

All matings with T2 females have fecundity = 1+f, where 0 <= f, while matings with T1 females have fecundity = 1

In[46]:=
```
m11f = FullSimplify[m11 * 1];
m12f = FullSimplify[m12 * 1];
m13f = FullSimplify[m13 * (1 + f)];
m14f = FullSimplify[m14 * (1 + f)];
m21f = FullSimplify[m21 * 1];
m22f = FullSimplify[m22 * 1];
m23f = FullSimplify[m23 * (1 + f)];
m24f = FullSimplify[m24 * (1 + f)];
m31f = FullSimplify[m31 * 1];
m32f = FullSimplify[m32 * 1];
m33f = FullSimplify[m33 * (1 + f)];
m34f = FullSimplify[m34 * (1 + f)];
m41f = FullSimplify[m41 * 1];
m42f = FullSimplify[m42 * 1];
m43f = FullSimplify[m43 * (1 + f)];
m44f = FullSimplify[m44 * (1 + f)];
```

Normalize by fb, which is the mean fecundity across the population

In[62]:=
```
fb = FullSimplify[1 * (m11 + m12 + m21 + m22 + m31 + m32 + m41 + m42) +
    (1 + f) * (m13 + m14 + m23 + m24 + m33 + m34 + m43 + m44)];
```

Normalized matings are thus:

```
In[63]:= no11 = FullSimplify[m11f / fb];
no12 = FullSimplify[m12f / fb];
no13 = FullSimplify[m13f / fb];
no14 = FullSimplify[m14f / fb];
no21 = FullSimplify[m21f / fb];
no22 = FullSimplify[m22f / fb];
no23 = FullSimplify[m23f / fb];
no24 = FullSimplify[m24f / fb];
no31 = FullSimplify[m31f / fb];
no32 = FullSimplify[m32f / fb];
no33 = FullSimplify[m33f / fb];
no34 = FullSimplify[m34f / fb];
no41 = FullSimplify[m41f / fb];
no42 = FullSimplify[m42f / fb];
no43 = FullSimplify[m43f / fb];
no44 = FullSimplify[m44f / fb];
```

Table format

```
In[79]:= ClearAll[F]

In[80]:= F = Table[0, {i, 4}, {j, 4}]

Out[80]= {{0, 0, 0, 0}, {0, 0, 0, 0}, {0, 0, 0, 0}, {0, 0, 0, 0}}
```

In[81]:=
```
F[[1, 1]] = no11;
F[[1, 2]] = no12;
F[[1, 3]] = no13;
F[[1, 4]] = no14;
F[[2, 1]] = no21;
F[[2, 2]] = no22;
F[[2, 3]] = no23;
F[[2, 4]] = no24;
F[[3, 1]] = no31;
F[[3, 2]] = no32;
F[[3, 3]] = no33;
F[[3, 4]] = no34;
F[[4, 1]] = no41;
F[[4, 2]] = no42;
F[[4, 3]] = no43;
F[[4, 4]] = no44;
```

In[97]:=
```
F;
```

In[98]:=
```
FullSimplify[F[[1, 1]] + F[[1, 2]] + F[[1, 3]] + F[[1, 4]] +
  F[[2, 1]] + F[[2, 2]] + F[[2, 3]] + F[[2, 4]] + F[[3, 1]] + F[[3, 2]] +
  F[[3, 3]] + F[[3, 4]] + F[[4, 1]] + F[[4, 2]] + F[[4, 3]] + F[[4, 4]]]
```

Out[98]= 1

Recursions

Recursions in terms of genotypes:

In[99]:=
```
ClearAll[r]
```

```
In[100]:= xt1[1] = F[[1, 1]] + (1/2) F[[1, 2]] + (1/2) F[[1, 3]] +
      (1/2) (1 - r) F[[1, 4]] + (1/2) F[[2, 1]] + (1/2) r F[[2, 3]] +
      (1/2) F[[3, 1]] + (1/2) r F[[3, 2]] + (1/2) (1 - r) F[[4, 1]];

In[101]:= xt1[2] = (1/2) F[[1,2]] + (1/2) r F[[1,4]] +
      (1/2) F[[2,1]] + F[[2,2]] + (1/2)(1-r)F[[2,3]] +
      (1/2) F[[2,4]] + (1/2) (1-r) F[[3,2]] +
      (1/2) r F[[4,1]] + (1/2) F[[4,2]];

In[102]:= xt1[3] = (1/2) F[[1,3]] + (1/2) r F[[1,4]] +
      (1/2) (1-r) F[[2,3]] + (1/2) F[[3,1]] +
      (1/2) (1-r) F[[3,2]] + F[[3,3]] + (1/2) F[[3,4]] +
      (1/2) r F[[4,1]] + (1/2) F[[4,3]];

In[103]:= xt1[4] =
   (1/2) (1-r) F[[1,4]] + (1/2) r F[[2,3]] +
      (1/2) F[[2,4]] + (1/2) r F[[3,2]] + (1/2) F[[3,4]] +
      (1/2) (1-r) F[[4,1]] + (1/2) F[[4,2]] + (1/2) F[[4,3]] + F[[4,4]];
```

In terms of allele frequencies:

```
In[105]:= x1 = (1 - t2) * (1 - p2) + diseq;
   x2 = (1 - t2) * p2 - diseq;
   x3 = t2 * (1 - p2) - diseq;
   x4 = t2 * p2 + diseq;
```

Frequencies in generation t+1: p2t1, t2t1, and diseqt1

If c=0, use FullSimplify for p2t1, Simplify for t2t1, and neither for diseqt1.

```
In[109]:= p2t1 = FullSimplify[xt1[2] + xt1[4]]

Out[109]= (p2 (1 + s t2 (-1 + μ)) (2 + 2 s t2 (-1 + μ) + f (ρ + t2 (-1 + μ) (-2 + 2 s + ρ))) +
     diseq (-1 + μ)
      (2 s (1 + s t2 (-1 + μ)) + f (-1 + s (1 + ρ + t2 (-1 + μ) (-2 + 2 s + ρ))))) /
   (2 (1 + s t2 (-1 + μ)) (1 + (f (-1 + s) + s) t2 (-1 + μ)))

In[110]:= t2t1 = Simplify[xt1[3] + xt1[4]]

Out[110]= ((-1 + s) (-1 + μ) ((-1 + p2) s (2 s + f (-1 + 2 s)) t2^3 (-1 + μ)^2 +
        t2^2 (-1 + μ) (2 s (-2 + 2 p2 + diseq s (-1 + μ)) +
           f (1 + p2 (-1 + 3 s) + 2 diseq s^2 (-1 + μ) + s (-3 + diseq - diseq μ))) +
        t2 (2 (-1 + p2 + diseq s (-1 + μ)) + f (-1 + p2 + diseq (-1 + μ) (s - ρ))) -
        diseq f ρ)) /
   (2 (1 + s t2 (-1 + μ)) (1 + f (-1 + s) t2 (-1 + μ) + s t2 (-1 + μ))
     (-1 + s t2 + p2 (1 + s t2 (-1 + μ)) + diseq s (-1 + μ) - s t2 μ))

In[111]:= diseqt1 = xt1[1] xt1[4] - xt1[2] xt1[3];
```

B. Analysis of the model

The influence of the parameters on the preference and trait frequencies

How does p2 change with the parameters?

1. How does f influence preference frequency?

In[112]:=
```
δp2f = FullSimplify[D[p2t1, f]]
```

Out[112]=
$$\left(p2\,\rho + (-1+\mu)\,(\mathrm{diseq}\,(-1+s) + (p2\,t2 + s\,(\mathrm{diseq}+p2\,t2)\,(1+t2\,(-1+\mu)))\,\rho)\right) \Big/ \left(2\,(1+(f\,(-1+s)+s)\,t2\,(-1+\mu))^2\right)$$

In[113]:=
```
FullSimplify[Reduce[δp2f > 0 && f > 0 &&
    0 < ρ < 1 && 0 < s < 1 && 0 < p2 < t2 < 1 && diseq ≥ 0 && 1 > μ ≥ 0]]
```

Out[113]=
$$0 \le \mu < 1 \;\&\&\; t2 < 1 \;\&\&\; f > 0 \;\&\&\; 0 < p2 < t2 \;\&\&\; \left(\left(\mathrm{diseq} \ge 0 \;\&\&\; \rho > 0 \;\&\&\; \left(\left(s > 0 \;\&\&\; \rho < 1 \;\&\&\; s \le \frac{1}{2+t2\,(-1+\mu)}\right) \;||\; \left(s < 1 \;\&\&\; \frac{1}{2+t2\,(-1+\mu)} < s \;\&\&\; \rho \le \frac{1-s}{s-s\,t2+s\,t2\,\mu}\right)\right)\right) \;||\; \left(0 \le \mathrm{diseq} < -((p2\,(1+t2\,(-1+\mu)))\,(1+s\,t2\,(-1+\mu))\,\rho) \,/\, ((-1+\mu)\,(-1+s+s\,(1+t2\,(-1+\mu))\,\rho))) \;\&\&\; \frac{1}{2+t2\,(-1+\mu)} < s < 1 \;\&\&\; \frac{1-s}{s-s\,t2+s\,t2\,\mu} < \rho < 1\right)\right)$$

Is the condition $\frac{1-s}{s-s\,t2+s\,t2\,\mu}$ possible (i.e., can ρ be greater than or less than this quantity)?

In[137]:=
```
FindMinimum[{(1 - s)/(s - s t2 + s t2 μ), 0 < s < 1 && 0 < μ < 1 && 0 < t2 < 1},
  {{s, 0.001}, {μ, 0.001}, {t2, 0.001}}]
```

FindMinimum::eit :

The algorithm does not converge to the tolerance of 4.806217383937354`*^-6 in 500 iterations. The best estimated solution, with feasibility residual, KKT residual, or complementary residual of $\{9.2226\times10^{-13}, 0.000128012, 2.93169\times10^{-13}\}$, is returned. ≫

Out[137]= {0.102265, {s → 0.912543, μ → 0.814941, t2 → 0.339617}}

In[136]:=
```
FindMaximum[{(1 - s)/(s - s t2 + s t2 μ), 0 < s < 1 && 0 < μ < 1 && 0 < t2 < 1},
  {{s, 0.001}, {μ, 0.001}, {t2, 0.001}}]
```

FindMaximum::eit :

The algorithm does not converge to the tolerance of 4.806217383937354`*^-6 in 500 iterations. The best estimated solution, with feasibility residual, KKT residual, or complementary residual of $\{1.3691\times10^{-9}, 1.22497\times10^{17}, 3.08682\times10^{-10}\}$, is returned. ≫

Out[136]= $\{3.50369\times10^{10}, \{s\to2.85738\times10^{-11}, \mu\to0.00113752, t2\to0.00113866\}\}$

Yes, this condition is relevant: ρ can be greater than the minimum and less than the maximum.

Is the condition

```
diseq < -((p2 (1 + t2 (-1 + μ)) (1 + s t2 (-1 + μ)) ρ) /
    ((-1 + μ) (-1 + s + s (1 + t2 (-1 + μ)) ρ)))
```

possible (can diseq be less than this quantity)?

```
In[139]:= FindMinimum[{-((p2 (1 + t2 (-1 + μ)) (1 + s t2 (-1 + μ)) ρ) /
        ((-1 + μ) (-1 + s + s (1 + t2 (-1 + μ)) ρ))),
     0 < s < 1 && 0 < μ < 1 && 0 < t2 < 1 && 0 < p2 < 1 && 0 < ρ < 1},
    {{s, 0.001}, {μ, 0.001}, {t2, 0.001}, {p2, 0.001}, {ρ, 0.001}}]
```

FindMinimum::eit :

The algorithm does not converge to the tolerance of 4.806217383937354`*^−6 in 500 iterations. The best estimated solution, with feasibility residual, KKT residual, or complementary residual of $\{1.23564\times10^{-8}, 3.44271\times10^{16}, 3.83499\times10^{-9}\}$, is returned. >>

```
Out[139]= {-1.16442×10^8, {s → 0.595045, μ → 0.370394,
    t2 → 0.0841672, p2 → 0.632266, ρ → 0.718625}}
```

```
In[140]:= FindMaximum[{-((p2 (1 + t2 (-1 + μ)) (1 + s t2 (-1 + μ)) ρ) /
        ((-1 + μ) (-1 + s + s (1 + t2 (-1 + μ)) ρ))),
     0 < s < 1 && 0 < μ < 1 && 0 < t2 < 1 && 0 < p2 < 1 && 0 < ρ < 1},
    {{s, 0.001}, {μ, 0.001}, {t2, 0.001}, {p2, 0.001}, {ρ, 0.001}}]
```

```
Out[140]= {-3.97948×10^-11, {s → 0.408574, μ → 0.381039,
    t2 → 0.293184, p2 → 0.0364918, ρ → 5.26764×10^-10}}
```

In[223]:=
```
(p2 (1 + t2 (-1 + μ)) (1 + s t2 (-1 + μ)) ρ) /
    ((-1 + μ) (-1 + s + s (1 + t2 (-1 + μ)) ρ)) /. p2 → 0.7 /.
  t2 → 0.8 /. μ → 0.001 /. s → 0.2 /. ρ → 0.3
```

Out[223]= 0.045007

Yes, this condition is relevant.

The analysis above shows that there are three relevant conditions. Preference frequency increases with f when:

a) $s \leq \frac{1}{2+t2\,(-1+\mu)}$, OR

b) $\frac{1}{2+t2\,(-1+\mu)} < s$ and $\rho \leq \frac{1-s}{s-s\,t2+s\,t2\,\mu}$, OR

c) $0 \leq \text{diseq} < -((p2\,(1+t2\,(-1+\mu))\,(1+s\,t2\,(-1+\mu))\,\rho)\,/\,((-1+\mu)\,(-1+s+s\,(1+t2\,(-1+\mu))\,\rho)))$ and $\frac{1}{2+t2\,(-1+\mu)} < s < 1$ and $\frac{1-s}{s-s\,t2+s\,t2\,\mu} < \rho < 1$

For (a), the whole fraction increases as t2 increases and μ decreases. Thus, the condition is more likely to be met when t2 is large and μ is small.

For (b), the ρ condition increases as s decreases, t2 increases, and μ decreases.

For (c), the conditions for s is the same as for (b), but the condition for ρ is opposite.

One of $s \leq \frac{1}{2+t2\,(-1+\mu)}$ and $\frac{1}{2+t2\,(-1+\mu)} < s$ is always true.

In (b), preference frequency will always increase with f as long as $\rho \le \frac{1-s}{s-s\,t2+s\,t2\,\mu}$. (c) requires $\frac{1-s}{s-s\,t2+s\,t2\,\mu} < \rho$.

ρ cannot be larger than 1, so when $\frac{1-s}{s-s\,t2+s\,t2\,\mu}$ is > 1, the conditions for case (b) will always be met, and the conditions for case (c) will never be met.

In[190]:= FullSimplify[Reduce[$\frac{1-s}{s-s\,t2+s\,t2\,\mu} > 1$ && 0 < s < 1 && 0 < t2 < 1 && 0 < μ < 1]]

Out[190]= $0 < \mu < 1$ && $0 < t2 < 1$ && $0 < s < \frac{1}{2 + t2\,(-1+\mu)}$

In[191]:= FindMinimum[{ $\frac{1}{2 + t2\,(-1+\mu)}$, 0 < μ < 1 && 0 < t2 < 1},
{{μ, 0.001}, {t2, 0.001}}]

Out[191]= {0.5, {μ → 0.50229, t2 → 6.09675×10^{-7}}}

In[192]:= FindMaximum[{ $\frac{1}{2 + t2\,(-1+\mu)}$, 0 < μ < 1 && 0 < t2 < 1},
{{μ, 0.001}, {t2, 0.001}}]

Out[192]= {1., {μ → 5.96859×10^{-9}, t2 → 1.}}

The condition for ρ in (b) is always true, and always false in (c), for s < 0.5. Because s > 0.5 seems very high, case (c) is unlikely to occur.

2. How does ρ influence preference frequency?

In[114]:= δp2ρ = FullSimplify[D[p2t1, ρ]]

Out[114]= (f (p2 (1 + s t2 (-1 + μ)) + diseq s (-1 + μ)) (1 + t2 (-1 + μ))) /
(2 (1 + s t2 (-1 + μ)) (1 + (f (-1 + s) + s) t2 (-1 + μ)))

In[115]:=
```
FullSimplify[Reduce[δp2ρ > 0 && f > 0 &&
    0 < ρ < 1 && 0 < s < 1 && 0 < p2 < t2 < 1 && diseq ≥ 0 && 1 > μ ≥ 0]]
```

Out[115]=
```
ρ > 0 && μ ≥ 0 && s > 0 && p2 > 0 && diseq ≥ 0 && f > 0 && ρ < 1 && μ < 1 &&
  t2 < 1 && s < 1 && p2 < t2 && s (diseq + p2 t2) < p2 + s (diseq + p2 t2) μ
```

Preference frequency increases with ρ when the following condition holds: s*(diseq+p2*t2)<p2+s*(diseq+p2*t2)*μ.
In terms of s, this condition simplifies to s < p2 / ((diseq + p2*t2) * (1-μ)).
Note that diseq+p2*t2 is equal to x4, the frequency of P2T2 genotypes. This frequency must be less than or equal to p2. Since 1-μ must be less than or equal to 1, this means that p2 is always equal to or greater than ((diseq + p2*t2) * (1-μ)). This means that p2 / ((diseq + p2*t2) * (1-μ)) will always be greater than or equal to 1, and thus will always be larger than s. This means that the condition always holds true, and thus that p2t1 will always increase with ρ.

3. How does s influence preference frequency?

In[116]:=
```
δp2s = FullSimplify[D[p2t1, s]]
```

Out[116]=

$$\Big((-1+\mu)\,\big(-f\,(1+f)\,p2\,t2\,(1+t2\,(-1+\mu))\,(1+s\,t2\,(-1+\mu))^2\,\rho + \text{diseq}\,\big(2\,(1+s\,t2\,(-1+\mu))^2 - f\,(1+s\,t2\,(-1+\mu))\,(-1-\rho + t2\,(-1+\mu)\,(2-3\,s+(-1+s-s\,t2+s\,t2\,\mu)\,\rho)) + f^2\,t2\,(-1+\mu)\,(-\rho+t2\,(-1+\mu)\,(1-\rho+s\,(-2+s+s\,(-1+t2-t2\,\mu)\,\rho)))\big)\big)\Big) \Big/ \Big(2\,(1+s\,t2\,(-1+\mu))^2\,(1+(f\,(-1+s)+s)\,t2\,(-1+\mu))^2\Big)$$

When does preference frequency decrease with s?

In[170]:=
```
FullSimplify[Reduce[δp2s < 0 && f > 0 &&
   0 < ρ < 1 && 0 < s < 1 && 0 < p2 < t2 < 1 && diseq ≥ 0 && 1 > μ ≥ 0]]
```

Out[170]=
```
0 ≤ μ < 1 && t2 < 1 && 0 < s < 1 && f > 0 && 0 < ρ < 1 && 0 < p2 < t2 &&
 diseq > -((f (1 + f) p2 t2 (1 + t2 (-1 + μ)) (1 + s t2 (-1 + μ))^2 ρ) /
     (-2 (1 + s t2 (-1 + μ))^2 + f (1 + s t2 (-1 + μ))
        (-1 - ρ + t2 (-1 + μ) (2 - ρ + s (-3 + ρ - t2 ρ + t2 μ ρ))) + f^2 t2
        (-1 + μ) (ρ + t2 (-1 + μ) (-1 + ρ + s (2 + s (-1 + ρ - t2 ρ + t2 μ ρ))))))
```

In[171]:=
```
diseqthreshold =
  FullSimplify[-((f (1 + f) p2 t2 (1 + t2 (-1 + μ)) (1 + s t2 (-1 + μ))^2 ρ) /
      (-2 (1 + s t2 (-1 + μ))^2 + f (1 + s t2 (-1 + μ))
         (-1 - ρ + t2 (-1 + μ) (2 - ρ + s (-3 + ρ - t2 ρ + t2 μ ρ))) + f^2 t2 (-1 + μ)
         (ρ + t2 (-1 + μ) (-1 + ρ + s (2 + s (-1 + ρ - t2 ρ + t2 μ ρ))))))];
```

In[172]:=
```
FindMinimum[{diseqthreshold,
   0 < f < 1 && 0 < s < 1 && 0 < μ < 1 && 0 < t2 < 1 && 0 < p2 < 1 && 0 < ρ < 1},
  {{s, 0.001}, {μ, 0.001}, {t2, 0.001},
   {p2, 0.001}, {ρ, 0.001}, {f, 0.001}}]
```

Out[172]=
```
{8.98246×10^-15, {s → 0.208707, μ → 0.205343, t2 → 0.0836925,
  p2 → 0.0348955, ρ → 0.0388065, f → 1.69806×10^-10}}
```

In[173]:=
```
FindMaximum[{diseqthreshold,
   0 < f < 1 && 0 < s < 1 && 0 < μ < 1 && 0 < t2 < 1 && 0 < p2 < 1 && 0 < ρ < 1},
  {{s, 0.001}, {μ, 0.001}, {t2, 0.001},
   {p2, 0.001}, {ρ, 0.001}, {f, 0.001}}]
```

Out[173]=
```
{0.499999, {s → 0.431386, μ → 1., t2 → 1., p2 → 1., ρ → 0.999999, f → 1.}}
```

As linkage disequilibrium increases, it becomes more likely that

preference frequency will decrease with s. This makes sense because greater linkage disequilibrium between the preference and the trait means that s will have a larger indirect effect on the preference frequency. Note that linkage disequilibrium cannot exceed 0.25.

When does preference frequency increase with s?

In[174]:=
```
FullSimplify[Reduce[δp2s > 0 && f > 0 &&
    0 < ρ < 1 && 0 < s < 1 && 0 < p2 < t2 < 1 && diseq ≥ 0 && 1 > μ ≥ 0]]
```

Out[174]=
```
0 ≤ μ < 1 && t2 < 1 && 0 < s < 1 && f > 0 && 0 < ρ < 1 && 0 < p2 < t2 &&
  0 ≤ diseq < -((f (1 + f) p2 t2 (1 + t2 (-1 + μ)) (1 + s t2 (-1 + μ))^2 ρ) /
      (-2 (1 + s t2 (-1 + μ))^2 + f (1 + s t2 (-1 + μ))
          (-1 - ρ + t2 (-1 + μ) (2 - ρ + s (-3 + ρ - t2 ρ + t2 μ ρ))) + f^2 t2
          (-1 + μ) (ρ + t2 (-1 + μ) (-1 + ρ + s (2 + s (-1 + ρ - t2 ρ + t2 μ ρ))))))
```

In[175]:=
```
diseqthreshold2 =
  FullSimplify[-((f (1 + f) p2 t2 (1 + t2 (-1 + μ)) (1 + s t2 (-1 + μ))^2 ρ) /
      (-2 (1 + s t2 (-1 + μ))^2 + f (1 + s t2 (-1 + μ))
          (-1 - ρ + t2 (-1 + μ) (2 - ρ + s (-3 + ρ - t2 ρ + t2 μ ρ))) + f^2 t2 (-1 + μ)
          (ρ + t2 (-1 + μ) (-1 + ρ + s (2 + s (-1 + ρ - t2 ρ + t2 μ ρ))))))];
```

In[176]:=
```
Reduce[diseqthreshold == diseqthreshold2]
```

Out[176]=
```
True
```

Preference frequency increases with s when linkage disequilibrium between the preference and the trait is greater than the same condition.

How does t2 change with the parameters?

1. How does f influence trait frequency?

In[118]:=
```
δt2f = FullSimplify[D[t2t1, f]]
```

Out[118]=
```
((-1 + s) (1 + t2 (-1 + μ)) (-1 + μ)
   (t2 (-1 + p2 + diseq s (-1 + μ)) + (-1 + p2) s t2^2 (-1 + μ) - diseq ρ)) /
 (2 (-1 + p2 (1 + s t2 (-1 + μ)) + s (diseq - t2) (-1 + μ))
   (1 + (f (-1 + s) + s) t2 (-1 + μ))^2)
```

In[119]:=
```
Reduce[δt2f > 0 && f > 0 && 0 < ρ < 1 &&
  0 < s < 1 && 0 < p2 < t2 < 1 && diseq ≥ 0 && 1 > μ ≥ 0]
```

Out[119]=
```
0 ≤ μ < 1 && 0 < t2 < 1 && 0 < s < 1 && 0 < p2 < t2 && diseq ≥ 0 && 0 < ρ < 1 && f > 0
```

Trait frequency increases with f for all realistic ranges of the parameters and variables.

2. How does ρ influence trait frequency?

In[120]:=
```
δt2ρ = FullSimplify[D[t2t1, ρ]]
```

Out[120]=
```
-((diseq f (-1 + s) (1 + t2 (-1 + μ)) (-1 + μ)) /
   (2 (-1 + p2 (1 + s t2 (-1 + μ)) + s (diseq - t2) (-1 + μ))
     (1 + s t2 (-1 + μ)) (1 + (f (-1 + s) + s) t2 (-1 + μ))))
```

In[121]:=
```
Reduce[δt2ρ > 0 && f > 0 && 0 < ρ < 1 &&
  0 < s < 1 && 0 < p2 < t2 < 1 && diseq ≥ 0 && 1 > μ ≥ 0]
```

Out[121]=
```
0 < ρ < 1 && 0 < t2 < 1 && 0 < p2 < t2 && 0 ≤ μ < 1 && 0 < s < 1 && diseq > 0 && f > 0
```

Trait frequency increases with ρ for all realistic ranges of the

parameters and variables.

3. How does s influence trait frequency?

In[123]:=
```
δt2s = FullSimplify[D[t2t1 /. μ → 0, s]]
```

Out[123]=
```
-(((-1 + s) t2 (1 - s t2) (-1 + p2 - diseq s + s t2 - p2 s t2)
      (1 - (f (-1 + s) + s) t2) ((-1 + p2) t2 (-4 + 4 s t2 + f (-3 - t2 + 4 s t2)) +
        diseq (-2 + 4 s t2 + f (-1 - t2 + 4 s t2))) +
     (1 + f) (-1 + s) t2 (1 - s t2) (-1 + p2 - diseq s + s t2 - p2 s t2)
      (t2 (1 - p2 + diseq s + (-1 + p2) s t2)
          (-2 + 2 s t2 + f (-1 + (-1 + 2 s) t2)) + diseq f (-1 + t2) ρ) -
     (-1 + s) (-diseq + t2 - p2 t2) (1 - s t2) (1 - (f (-1 + s) + s) t2)
      (t2 (1 - p2 + diseq s + (-1 + p2) s t2)
          (-2 + 2 s t2 + f (-1 + (-1 + 2 s) t2)) + diseq f (-1 + t2) ρ) +
     (-1 + s) t2 (-1 + p2 - diseq s + s t2 - p2 s t2) (1 - (f (-1 + s) + s) t2)
      (t2 (1 - p2 + diseq s + (-1 + p2) s t2)
          (-2 + 2 s t2 + f (-1 + (-1 + 2 s) t2)) + diseq f (-1 + t2) ρ) +
     (1 - s t2) (-1 + p2 - diseq s + s t2 - p2 s t2) (1 - (f (-1 + s) + s) t2)
      (t2 (1 - p2 + diseq s + (-1 + p2) s t2)
          (-2 + 2 s t2 + f (-1 + (-1 + 2 s) t2)) + diseq f (-1 + t2) ρ)) /
   (2 (-1 + s t2)^2 (-1 + f (-1 + s) t2 + s t2)^2
     (1 - p2 + diseq s + (-1 + p2) s t2)^2))
```

In[124]:=
```
Reduce[δt2s < 0 && f > 0 && 0 < ρ < 1 &&
   0 < s < 1 && 0 < p2 < t2 < 1 && diseq ≥ 0 && 1 > μ ≥ 0]
```

Out[124]=
```
0 ≤ μ < 1 && 0 < s < 1 && 0 < t2 < 1 && f > 0 && 0 < p2 < t2 && diseq ≥ 0 && 0 < ρ < 1
```

Trait frequency decreases with s for all realistic ranges of the parameters and variables.

Evolution in the absence of the aversion (ρ = 0)

In[196]:= `p2ρ0 = FullSimplify[p2t1 /. ρ → 0]`

Out[196]= $p2 + \frac{1}{2\,t2} diseq \left(2 - \frac{1}{1 + (f\,(-1+s)+s)\,t2\,(-1+\mu)} + \frac{1}{-1 + s\,t2 - s\,t2\,\mu}\right)$

In[197]:= `t2ρ0 = FullSimplify[t2t1 /. ρ → 0]`

Out[197]= $((-1+s)\,t2\,(2+f\,(1+(-1+2\,s)\,t2\,(-1+\mu))+2\,s\,t2\,(-1+\mu))\,(-1+\mu))\,/$
$(2\,(1+s\,t2\,(-1+\mu))\,(1+(f\,(-1+s)+s)\,t2\,(-1+\mu)))$

In[198]:= `diseqρ0 = FullSimplify[diseqt1 /. ρ → 0]`

Out[198]= $-\Big(\big(diseq\,(-1+s)\,(-1+\mu)\,\big(4\,(-1+r)\,(1+s\,t2\,(-1+\mu))^2 +$
$2\,f\,(-1+r)\,(1+s\,t2\,(-1+\mu))\,(1+(-2+3\,s)\,t2\,(-1+\mu)) + f^2\,(-1+s)$
$t2\,(-1+\mu)\,(-1+2\,r\,(1+s\,t2\,(-1+\mu))+t2\,(-1+2\,s+\mu-2\,s\,\mu))\big)\big)\,/$
$\big(4\,(1+s\,t2\,(-1+\mu))^2\,(1+(f\,(-1+s)+s)\,t2\,(-1+\mu))^2\big)\Big)$

1. Will p2 increase with s when ρ=0?

`dp2ρ0s = FullSimplify[D[p2ρ0, s]]`

$\frac{1}{2} diseq \left(\frac{1}{(1+s\,t2\,(-1+\mu))^2} + \frac{1+f}{(1+(f\,(-1+s)+s)\,t2\,(-1+\mu))^2}\right)(-1+\mu)$

`Reduce[dp2ρ0s > 0 && diseq ≥ 0 && 0 ≤ t2 ≤ 1 && 0 < f < 1 && 0 < s < 1 && 0 ≤ μ < 1]`

`False`

p2 will not increase with s when ρ=0.

2. Will p2 increase with f when ρ=0?

```
dp2ρ0f = FullSimplify[D[p2ρ0, f]]
```

$$\frac{\text{diseq}\,(-1+s)\,(-1+\mu)}{2\,(1+(f\,(-1+s)+s)\,t2\,(-1+\mu))^2}$$

```
Reduce[dp2ρ0f > 0 && diseq ≥ 0 && 0 ≤ t2 ≤ 1 && 0 < f < 1 && 0 < s < 1 && 0 ≤ μ < 1]
```

0 < s < 1 && 0 < f < 1 && 0 ≤ μ < 1 && 0 ≤ t2 ≤ 1 && diseq > 0

p2 will only increase with f when ρ=0 if diseq is greater than 0.

3. If diseqt1 = 0 when diseq = 0 and ρ = 0, we can conclude that linkage disequilibrium cannot build up between the preference and the trait in the absence of the aversion. Does diseqt1 = 0 when diseq = 0 and ρ = 0?

In[199]:=
```
diseqρ0 /. diseq → 0
```

Out[199]= 0

Yes.

Conclusion: when ρ=0, p2 will not increase with s under any conditions, and p2 will only increase with f when diseq > 0. However, because our starting diseq in the simulations is 0, p2 will never increase when f is increasing in the simulations, as long as ρ=0. This demonstrates both the importance of the aversion, and the

importance of linkage disequilibrium between the preference and the trait, for the evolution of the preference. Further, we have shown that linkage disequilibrium does not build up between the preference and the trait in the absence of the aversion.

Does sexual selection occur in the model?

If sexual selection occurs, aversion strength alone will influence p2 and t2. Test this by setting s and f equal to zero, and determining whether aversion strength influences the allele frequencies.

```
In[193]:= FullSimplify[p2t1 /. s → 0 /. f → 0]
Out[193]= p2
```

```
In[194]:= FullSimplify[t2t1 /. s → 0 /. f → 0]
Out[194]= t2 - t2 μ
```

In the absence of s and f, neither p2t1 nor t2t1 is influenced by the aversion (ρ).

When will t2 persist in the population with no preference?

Calculated using c = 0

Goal: find the conditions under which Δt2 is positive when p2=0 (and thus diseq=0). This should tell us when we expect t2 to persist in the absence of a preference.

In[114]:=
```
Δt2 = FullSimplify[t2t1 - t2]
```

Out[114]=
```
-t2 + ((-1 + s) (-1 + μ) (t2 (-1 + p2 (1 + s t2 (-1 + μ)) + s (diseq - t2) (-1 + μ))
        (2 + f (1 + (-1 + 2 s) t2 (-1 + μ)) + 2 s t2 (-1 + μ)) +
      diseq f (-1 + t2 - t2 μ) ρ)) /
   (2 (-1 + p2 (1 + s t2 (-1 + μ)) + s (diseq - t2) (-1 + μ))
     (1 + s t2 (-1 + μ)) (1 + (f (-1 + s) + s) t2 (-1 + μ)))
```

In[124]:=
```
Δt2p20 = FullSimplify[Δt2 /. p2 → 0 /. diseq → 0]
```

Out[124]=

$$\frac{1}{2}\,t2\left(-2+((-1+s)\,(2+f\,(1+(-1+2s)\,t2\,(-1+\mu))+2\,s\,t2\,(-1+\mu))\,(-1+\mu))\,/\,((1+s\,t2\,(-1+\mu))\,(1+(f\,(-1+s)+s)\,t2\,(-1+\mu)))\right)$$

Note that c does not appear in the expression for Δt2 when p2=0, indicating that further analyses apply equally to cases with and without a cost.

Under what conditions is Δt2 positive (meaning that we can expect t2 to persist in the population) when p2=0?

In[118]:=
```
FullSimplify[
 Reduce[Δt2p20 > 0 && 0 < s < 1 && 0 < t2 < 1 && 0 < f < 1 && 0 < μ < 1]]
```

Out[118]=
$$0 < \mu < \frac{1}{3} \;\&\&\; 0 < t2 < \frac{-1+3\mu}{-1+\mu^2} \;\&\&\; s > 0 \;\&\&\; 2 + \frac{3}{t2 - t2\,\mu} > \frac{8\,s + \sqrt{\left(\left((9+4\,(-1+t2)\,t2)\,(1+t2\,(-1+\mu))^2\right) \big/ \left((-1+t2)^2\,t2^2\,(-1+\mu)^2\right)\right)} + 3\,\mu}{(-1+t2)\,(-1+\mu)} \;\&\&\; -((2\,(1+s\,t2\,(-1+\mu))\,(s\,(-1+t2)\,(-1+\mu)+\mu)) \,/\, ((-1+s)\,(-1+\mu)\,(-1+t2\,(1+2\,s\,(-1+t2)\,(-1+\mu)+\mu)))) < f < 1$$

1. What is the condition for Δt2 to be positive when s=0.1 and *μ*=0.01, as in our simulations shown in Figures 1A, 2A, and 2B?

In[127]:=
```
Δt2p201 = FullSimplify[Δt2p20 /. s → 0.1 /. μ → 0.01]
```

Out[127]=
$$\frac{1}{2}\,t2\,(-2 + (0.891\,(2 + f - 0.198\,t2 + 0.792\,f\,t2)) \,/\, ((1 - 0.099\,t2)\,(1 - 0.099\,t2 + 0.891\,f\,t2)))$$

In[128]:=
```
FullSimplify[Reduce[Δt2p201 > 0 && 0 ≤ f ≤ 1 && 0 < t2 ≤ 1]]
```

Reduce::ratnz :

Reduce was unable to solve the system with inexact coefficients. The answer was obtained by solving a corresponding exact system and numericizing the result. ≫

Out[128]=
$$0 < t2 < 0.951106 \;\&\&\; (1.2357 + (-1.24467 + 0.111111\,t2)\,t2) \,/\, (5.05051 + t2\,(-6.10101 + 1.\,t2)) < f \le 1.$$

In[137]:=
```
fthreshold1 = (1.2357015724019094` +
      (-1.2446689113355782` + 0.11111111111111112` t2) t2) /
    (5.05050505050505` + t2 (-6.101010101010101` + 1.` t2));
```

Δt2 is positive when t2 < 0.951106 and f > $\frac{1.2357+(-1.24467+0.111111\,t2)\,t2}{5.05051+t2\,(-6.10101+1.\,t2)}$. This means that, for the conditions s=0.1 and μ=0.01, when p2=0, t2 will increase as long as t2 < 0.951106 and f meets the condition above. Plugging in the starting values we use in our simulations (t2=0.8 in the simulations shown in the main text, and t2=0.1 in the simulations shown in the supplementary figures) gives the threshold value of f above which t2 will persist in the absence of p2.

In[138]:=
```
fthreshold1 /. t2 → 0.1
fthreshold1 /. t2 → 0.8
```

Out[138]= 0.249943

Out[139]= 0.38419

These values are displayed as dashed lines on the appropriate figures (1A, 2A, and 2B in the main text; supplementary figure 1), showing the value of f above which t2 is expected to persist in the absence of a preference.

2. What is the condition for Δt2 to be positive when s=0.2 and μ=0.01, as in our simulations shown in Figure 1B?

In[133]:=
```
Δt2p202 = FullSimplify[Δt2p20 /. s → 0.2 /. μ → 0.01]
```

Out[133]=

$$\frac{1}{2}\,t2\,\left(-2+\left(0.792\,(2+f-0.396\,t2+0.594\,f\,t2)\right)/\left((1-0.198\,t2)\,(1-0.198\,t2+0.792\,f\,t2)\right)\right)$$

In[134]:=
```
FullSimplify[Reduce[Δt2p202 > 0 && 0 ≤ f ≤ 1 && 0 < t2 ≤ 1]]
```

Reduce::ratnz :

Reduce was unable to solve the system with inexact coefficients. The answer was obtained by solving a corresponding exact system and numericizing the result. >>

Out[134]=

$$0 < t2 < 0.876377\ \&\&\ \frac{1.3264+(-1.52525+0.25\,t2)\,t2}{2.52525+t2\,(-3.55051+1.\,t2)} < f \le 1.$$

In[136]:=
```
fthreshold2 =
  (1.326395265789205` + (-1.525252525252525253` + 0.25` t2) t2) /
   (2.52525252525252525` + t2 (-3.5505050505050506` + 1.` t2));
```

Δt2 is positive when t2 < 0.876377 and f > $\frac{1.3264+(-1.52525+0.25\,t2)\,t2}{2.52525+t2\,(-3.55051+1.\,t2)}$. This means that, for the conditions s=0.2 and μ=0.01, when p2=0, t2 will increase as long as t2 < 0.876377 and f meets the condition above. Plugging in the starting

values we use in our simulations (t2=0.8 in the simulations shown in the main text, and t2=0.1 in the simulations shown in the supplementary figures) gives the threshold value of f above which t2 will persist in the absence of p2.

```
In[140]:= fthreshold2 /. t2 → 0.1
          fthreshold2 /. t2 → 0.8

Out[140]= 0.539569

Out[141]= 0.819438
```

These values are displayed as dashed lines on the appropriate figures (1B in the main text, and supplementary figure 2), showing the value of f above which t2 is expected to persist in the absence of a preference.

C. Simulations

For simulations

```
p2sims = p2t1;
t2sims = t2t1;
diseqsims = diseqt1;
```

Simulations

```
r = 0.5;
f = 0.6;
ρ = 0.001;
s = 0.1;
μ = 0.01;
p2 = 0.05;
t2 = 0.1;
diseq = 0;
j = 2000;

tabp2 = Table[0, {i, j}];
tabt2 = Table[0, {i, j}];
tabdiseq = Table[0, {i, j}];
Do[
  tabp2[[i]] = p2;
  tabt2[[i]] = t2;
  tabdiseq[[i]] = diseq;
  p2t1 = p2sims;
  t2t1 = t2sims;
  diseqt1 = diseqsims;
  p2 = p2t1;
  t2 = t2t1;
  diseq = diseqt1; If[p2 > t2, Break[]],
  {i, j}];
p2plot =
 ListPlot[tabp2, Joined → True, PlotRange → {{0, j}, {0.0, 1.0}}]
t2plot = ListPlot[tabt2, Joined → True,
  PlotRange → {{0, j}, {0.0, 1.0}}]
diseqplot = ListPlot[tabdiseq, Joined → True]
```

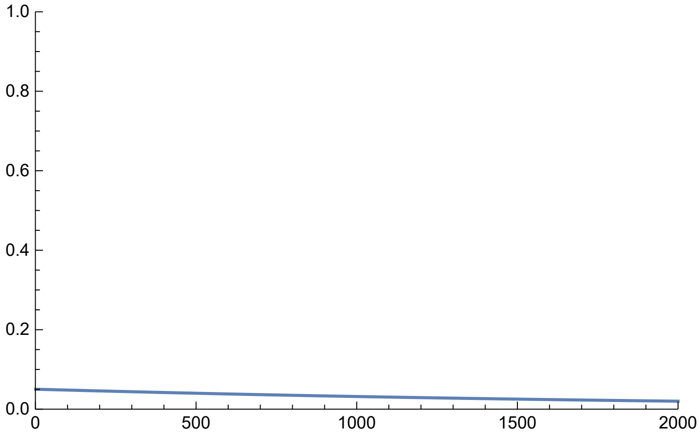
1.0
0.8
0.6
0.4
0.2
0.0
0
500
1000
1500
2000

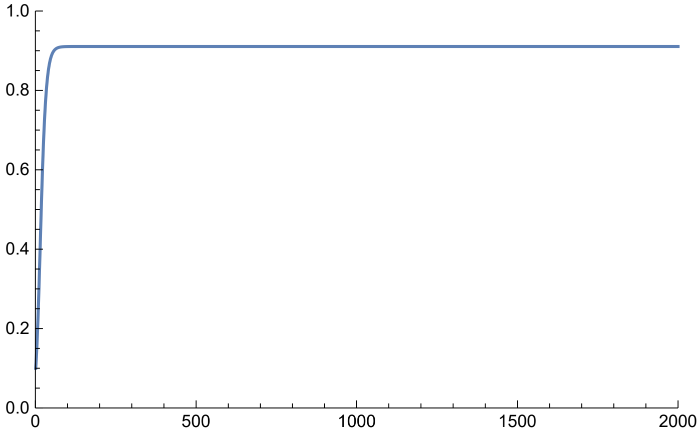
1.0
0.8
0.6
0.4
0.2
0.0
0
500
1000
1500
2000

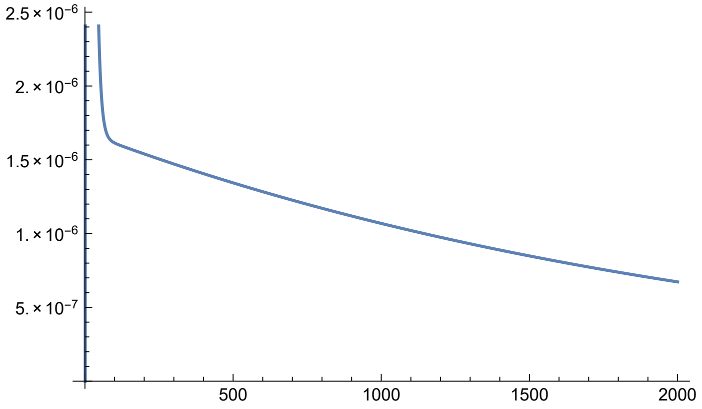

tabp2[[1900 ;; 2000]]

{0.0209519, 0.0209421, 0.0209323, 0.0209225, 0.0209127,
0.020903, 0.0208932, 0.0208834, 0.0208737, 0.0208639, 0.0208542,
0.0208444, 0.0208347, 0.020825, 0.0208152, 0.0208055, 0.0207958,
0.0207861, 0.0207764, 0.0207667, 0.020757, 0.0207473, 0.0207376,
0.0207279, 0.0207182, 0.0207085, 0.0206988, 0.0206892, 0.0206795,
0.0206698, 0.0206602, 0.0206505, 0.0206409, 0.0206312, 0.0206216,
0.020612, 0.0206023, 0.0205927, 0.0205831, 0.0205735, 0.0205638,
0.0205542, 0.0205446, 0.020535, 0.0205254, 0.0205158, 0.0205063,
0.0204967, 0.0204871, 0.0204775, 0.0204679, 0.0204584, 0.0204488,
0.0204393, 0.0204297, 0.0204202, 0.0204106, 0.0204011, 0.0203915,
0.020382, 0.0203725, 0.020363, 0.0203534, 0.0203439, 0.0203344,
0.0203249, 0.0203154, 0.0203059, 0.0202964, 0.0202869, 0.0202775,
0.020268, 0.0202585, 0.020249, 0.0202396, 0.0202301, 0.0202206,
0.0202112, 0.0202017, 0.0201923, 0.0201829, 0.0201734, 0.020164,
0.0201546, 0.0201451, 0.0201357, 0.0201263, 0.0201169, 0.0201075,
0.0200981, 0.0200887, 0.0200793, 0.0200699, 0.0200605, 0.0200511,
0.0200418, 0.0200324, 0.020023, 0.0200137, 0.0200043, 0.019995}

tabt2[[1 ;; 1000]]

{0.1, 0.116955, 0.136168, 0.157742, 0.181723, 0.208088, 0.236735,
0.267478, 0.300046, 0.334093, 0.369216, 0.404972, 0.440905, 0.476572,

```
0.511558, 0.5455, 0.578093, 0.609099, 0.638344, 0.665718, 0.691163,
0.714672, 0.736273, 0.756025, 0.774011, 0.790326, 0.805077,
0.818376, 0.830335, 0.841065, 0.850674, 0.859264, 0.866931,
0.873766, 0.879852, 0.885265, 0.890076, 0.894348, 0.898139, 0.9015,
0.90448, 0.90712, 0.909458, 0.911527, 0.913359, 0.914979, 0.916412,
0.917679, 0.9188, 0.91979, 0.920666, 0.921439, 0.922123, 0.922727,
0.92326, 0.923732, 0.924148, 0.924516, 0.92484, 0.925127, 0.92538,
0.925604, 0.925802, 0.925976, 0.92613, 0.926266, 0.926386, 0.926492,
0.926586, 0.926668, 0.926741, 0.926806, 0.926863, 0.926913,
0.926957, 0.926997, 0.927031, 0.927062, 0.927089, 0.927112,
0.927133, 0.927152, 0.927168, 0.927183, 0.927195, 0.927207,
0.927217, 0.927225, 0.927233, 0.92724, 0.927246, 0.927251, 0.927256,
0.92726, 0.927264, 0.927267, 0.92727, 0.927273, 0.927275, 0.927277,
0.927278, 0.92728, 0.927281, 0.927283, 0.927284, 0.927285, 0.927285,
0.927286, 0.927287, 0.927287, 0.927288, 0.927288, 0.927289,
0.927289, 0.927289, 0.92729, 0.92729, 0.92729, 0.92729, 0.92729,
0.92729, 0.927291, 0.927291, 0.927291, 0.927291, 0.927291, 0.927291,
0.927291, 0.927291, 0.927291, 0.927291, 0.927291, 0.927291,
0.927291, 0.927291, 0.927291, 0.927291, 0.927291, 0.927291, 0.927291,
0.927291, 0.927291, 0.927291, 0.927291, 0.927291, 0.927291,
0.927292, 0.927292, 0.927292, 0.927292, 0.927292, 0.927292, 0.927292,
0.927292, 0.927292, 0.927292, 0.927292, 0.927292, 0.927292,
0.927292, 0.927292, 0.927292, 0.927292, 0.927292, 0.927292, 0.927292,
0.927292, 0.927292, 0.927292, 0.927292, 0.927292, 0.927292,
0.927292, 0.927292, 0.927292, 0.927292, 0.927292, 0.927292,
0.927292, 0.927292, 0.927292, 0.927292, 0.927292, 0.927292, 0.927292,
0.927292, 0.927292, 0.927292, 0.927292, 0.927292, 0.927292, 0.927292,
0.927292, 0.927292, 0.927292, 0.927292, 0.927292, 0.927292,
0.927292, 0.927292, 0.927292, 0.927292, 0.927292, 0.927292, 0.927292,
0.927292, 0.927292, 0.927292, 0.927292, 0.927292, 0.927292, 0.927292,
0.927292, 0.927292, 0.927292, 0.927292, 0.927292, 0.927292, 0.927292,
0.927292, 0.927292, 0.927292, 0.927292, 0.927292, 0.927292, 0.927292,
```

0.927292, 0.927292, 0.927292, 0.927292, 0.927292, 0.927292, 0.927292,
0.927292, 0.927292, 0.927292, 0.927292, 0.927292, 0.927292, 0.927292,
0.927292, 0.927292, 0.927292, 0.927292, 0.927292, 0.927292, 0.927292,
0.927292, 0.927292, 0.927292, 0.927292, 0.927292, 0.927292, 0.927292,
0.927292, 0.927292, 0.927292, 0.927292, 0.927292, 0.927292, 0.927292,
0.927292, 0.927292, 0.927292, 0.927292, 0.927292, 0.927292, 0.927292,
0.927292, 0.927292, 0.927292, 0.927292, 0.927292, 0.927292, 0.927292,
0.927292, 0.927292, 0.927292, 0.927292, 0.927292, 0.927292, 0.927292,
0.927292, 0.927292, 0.927292, 0.927292, 0.927292, 0.927292, 0.927292,
0.927292, 0.927292, 0.927292, 0.927292, 0.927292, 0.927292, 0.927292,
0.927292, 0.927292, 0.927292, 0.927292, 0.927292, 0.927292, 0.927292,
0.927292, 0.927292, 0.927292, 0.927292, 0.927292, 0.927292, 0.927292,
0.927292, 0.927292, 0.927292, 0.927292, 0.927292, 0.927292, 0.927292,
0.927292, 0.927292, 0.927292, 0.927292, 0.927292, 0.927292, 0.927292,
0.927292, 0.927292, 0.927292, 0.927292, 0.927292, 0.927292, 0.927292,
0.927292, 0.927292, 0.927292, 0.927292, 0.927292, 0.927292, 0.927292,
0.927292, 0.927292, 0.927292, 0.927292, 0.927292, 0.927292, 0.927292,
0.927292, 0.927292, 0.927292, 0.927292, 0.927292, 0.927292, 0.927292,
0.927292, 0.927292, 0.927292, 0.927292, 0.927292, 0.927292, 0.927292,
0.927292, 0.927292, 0.927292, 0.927292, 0.927292, 0.927292, 0.927292,
0.927292, 0.927292, 0.927292, 0.927292, 0.927292, 0.927292, 0.927292,
0.927292, 0.927292, 0.927292, 0.927292, 0.927292, 0.927292, 0.927292,
0.927292, 0.927292, 0.927292, 0.927292, 0.927292, 0.927292, 0.927292,
0.927292, 0.927292, 0.927292, 0.927292, 0.927292, 0.927292, 0.927292,
0.927292, 0.927292, 0.927292, 0.927292, 0.927292, 0.927292, 0.927292,
0.927292, 0.927292, 0.927292, 0.927292, 0.927292, 0.927292, 0.927292,
0.927292, 0.927292, 0.927292, 0.927292, 0.927292, 0.927292, 0.927292,
0.927292, 0.927292, 0.927292, 0.927292, 0.927292, 0.927292, 0.927292,
0.927292, 0.927292, 0.927292, 0.927292, 0.927292, 0.927292, 0.927292,
0.927292, 0.927292, 0.927292, 0.927292, 0.927292, 0.927292, 0.927292,
0.927292, 0.927292, 0.927292, 0.927292, 0.927292, 0.927292, 0.927292,
0.927292, 0.927292, 0.927292, 0.927292, 0.927292, 0.927292, 0.927292,

```
0.927292, 0.927292, 0.927292, 0.927292, 0.927292, 0.927292, 0.927292,
0.927292, 0.927292, 0.927292, 0.927292, 0.927292, 0.927292, 0.927292,
0.927292, 0.927292, 0.927292, 0.927292, 0.927292, 0.927292, 0.927292,
0.927292, 0.927292, 0.927292, 0.927292, 0.927292, 0.927292, 0.927292,
0.927292, 0.927292, 0.927292, 0.927292, 0.927292, 0.927292, 0.927292,
0.927292, 0.927292, 0.927292, 0.927292, 0.927292, 0.927292, 0.927292,
0.927292, 0.927292, 0.927292, 0.927292, 0.927292, 0.927292, 0.927292,
0.927292, 0.927292, 0.927292, 0.927292, 0.927292, 0.927292, 0.927292,
0.927292, 0.927292, 0.927292, 0.927292, 0.927292, 0.927292, 0.927292,
0.927292, 0.927292, 0.927292, 0.927292, 0.927292, 0.927292, 0.927292,
0.927292, 0.927292, 0.927292, 0.927292, 0.927292, 0.927292,
0.927292, 0.927292, 0.927292, 0.927292, 0.927292, 0.927292,
0.927292, 0.927292, 0.927292, 0.927292, 0.927292, 0.927292,
0.927292, 0.927292, 0.927292, 0.927292, 0.927292, 0.927292,
0.927292, 0.927292, 0.927292, 0.927292, 0.927292, 0.927292,
0.927292, 0.927292, 0.927292, 0.927292, 0.927292, 0.927292,
0.927292, 0.927292, 0.927292, 0.927292, 0.927292, 0.927292,
0.927292, 0.927292, 0.927292, 0.927292, 0.927292, 0.927292,
0.927292, 0.927292, 0.927292, 0.927292, 0.927292, 0.927292,
0.927292, 0.927292, 0.927292, 0.927292, 0.927292, 0.927292,
0.927292, 0.927292, 0.927292, 0.927292, 0.927292, 0.927292,
0.927292, 0.927292, 0.927292, 0.927292, 0.927292, 0.927292,
0.927292, 0.927292, 0.927292, 0.927292, 0.927292, 0.927292,
0.927292, 0.927292, 0.927292, 0.927292, 0.927292, 0.927292,
0.927292, 0.927292, 0.927292, 0.927292, 0.927292, 0.927292,
0.927292, 0.927292, 0.927292, 0.927292, 0.927292, 0.927292,
0.927292, 0.927292, 0.927292, 0.927292, 0.927292, 0.927292,
0.927292, 0.927292, 0.927292, 0.927292, 0.927292, 0.927292,
0.927292, 0.927292, 0.927292, 0.927292, 0.927292, 0.927292,
0.927292, 0.927292, 0.927292, 0.927292, 0.927292, 0.927292,
0.927292, 0.927292, 0.927292, 0.927292, 0.927292, 0.927292,
0.927292, 0.927292, 0.927292, 0.927292, 0.927292, 0.927292,
```

```
0.927292, 0.927292, 0.927292, 0.927292, 0.927292, 0.927292,
0.927292, 0.927292, 0.927292, 0.927292, 0.927292, 0.927292,
0.927292, 0.927292, 0.927292, 0.927292, 0.927292, 0.927292,
0.927292, 0.927292, 0.927292, 0.927292, 0.927292, 0.927292,
0.927292, 0.927292, 0.927292, 0.927292, 0.927292, 0.927292,
0.927292, 0.927292, 0.927292, 0.927292, 0.927292, 0.927292,
0.927292, 0.927292, 0.927292, 0.927292, 0.927292, 0.927292,
0.927292, 0.927292, 0.927292, 0.927292, 0.927292, 0.927292,
0.927292, 0.927292, 0.927292, 0.927292, 0.927292, 0.927292,
0.927292, 0.927292, 0.927292, 0.927292, 0.927292, 0.927292,
0.927292, 0.927292, 0.927292, 0.927292, 0.927292, 0.927292,
0.927292, 0.927292, 0.927292, 0.927292, 0.927292, 0.927292,
0.927292, 0.927292, 0.927292, 0.927292, 0.927292, 0.927292,
0.927292, 0.927292, 0.927292, 0.927292, 0.927292, 0.927292,
0.927292, 0.927292, 0.927292, 0.927292, 0.927292, 0.927292,
0.927292, 0.927292, 0.927292, 0.927292, 0.927292, 0.927292,
0.927292, 0.927292, 0.927292, 0.927292, 0.927292, 0.927292,
0.927292, 0.927292, 0.927292, 0.927292, 0.927292, 0.927292,
0.927292, 0.927292, 0.927292, 0.927292, 0.927292, 0.927292,
0.927292, 0.927292, 0.927292, 0.927292, 0.927292, 0.927292,
0.927292, 0.927292, 0.927292, 0.927292, 0.927292, 0.927292,
0.927292, 0.927292, 0.927292, 0.927292, 0.927292, 0.927292,
0.927292, 0.927292, 0.927292, 0.927292, 0.927292, 0.927292,
0.927292, 0.927292, 0.927292, 0.927292, 0.927292, 0.927292,
0.927292, 0.927292, 0.927292, 0.927292, 0.927292, 0.927292,
0.927292, 0.927292, 0.927292, 0.927292, 0.927292, 0.927292,
0.927292, 0.927292, 0.927292, 0.927292, 0.927292, 0.927292,
0.927292, 0.927292, 0.927292, 0.927292, 0.927292, 0.927292,
0.927292, 0.927292, 0.927292, 0.927292, 0.927292, 0.927292,
0.927292, 0.927292, 0.927292, 0.927292, 0.927292, 0.927292,
0.927292, 0.927292, 0.927292, 0.927292, 0.927292, 0.927292,
0.927292, 0.927292, 0.927292, 0.927292, 0.927292, 0.927292,
```

```
0.927292, 0.927292, 0.927292, 0.927292, 0.927292, 0.927292,
0.927292, 0.927292, 0.927292, 0.927292, 0.927292, 0.927292,
0.927292, 0.927292, 0.927292, 0.927292, 0.927292, 0.927292,
0.927292, 0.927292, 0.927292, 0.927292, 0.927292, 0.927292,
0.927292, 0.927292, 0.927292, 0.927292, 0.927292, 0.927292,
0.927292, 0.927292, 0.927292, 0.927292, 0.927292, 0.927292,
0.927292, 0.927292, 0.927292, 0.927292, 0.927292, 0.927292,
0.927292, 0.927292, 0.927292, 0.927292, 0.927292, 0.927292,
0.927292, 0.927292, 0.927292, 0.927292, 0.927292, 0.927292,
0.927292, 0.927292, 0.927292, 0.927292, 0.927292, 0.927292,
0.927292, 0.927292, 0.927292, 0.927292, 0.927292, 0.927292,
0.927292, 0.927292, 0.927292, 0.927292, 0.927292, 0.927292,
0.927292, 0.927292, 0.927292, 0.927292, 0.927292, 0.927292,
0.927292, 0.927292, 0.927292, 0.927292, 0.927292, 0.927292,
0.927292, 0.927292, 0.927292, 0.927292, 0.927292, 0.927292,
0.927292, 0.927292, 0.927292, 0.927292, 0.927292, 0.927292,
0.927292, 0.927292, 0.927292, 0.927292, 0.927292, 0.927292,
0.927292, 0.927292, 0.927292, 0.927292, 0.927292, 0.927292,
0.927292, 0.927292, 0.927292, 0.927292, 0.927292, 0.927292,
0.927292, 0.927292, 0.927292, 0.927292, 0.927292, 0.927292,
0.927292, 0.927292, 0.927292, 0.927292, 0.927292, 0.927292,
0.927292, 0.927292, 0.927292, 0.927292, 0.927292, 0.927292,
0.927292, 0.927292, 0.927292, 0.927292, 0.927292, 0.927292,
0.927292, 0.927292, 0.927292, 0.927292, 0.927292, 0.927292,
0.927292, 0.927292, 0.927292, 0.927292, 0.927292, 0.927292,
0.927292, 0.927292, 0.927292, 0.927292, 0.927292, 0.927292}
```

```
tabdiseq[[1900 ;; 2000]]
```

{1.01047 × 10^-6, 1.01001 × 10^-6, 1.00954 × 10^-6, 1.00908 × 10^-6, 1.00861 × 10^-6, 1.00815 × 10^-6, 1.00768 × 10^-6, 1.00722 × 10^-6, 1.00675 × 10^-6, 1.00629 × 10^-6, 1.00582 × 10^-6, 1.00536 × 10^-6, 1.00489 × 10^-6, 1.00443 × 10^-6, 1.00397 × 10^-6, 1.0035 × 10^-6, 1.00304 × 10^-6, 1.00258 × 10^-6, 1.00212 × 10^-6, 1.00165 × 10^-6, 1.00119 × 10^-6, 1.00073 × 10^-6, 1.00027 × 10^-6, 9.99805 × 10^-7, 9.99344 × 10^-7, 9.98883 × 10^-7, 9.98422 × 10^-7, 9.97961 × 10^-7, 9.97501 × 10^-7, 9.97041 × 10^-7, 9.96581 × 10^-7, 9.96121 × 10^-7, 9.95661 × 10^-7, 9.95202 × 10^-7, 9.94742 × 10^-7, 9.94283 × 10^-7, 9.93824 × 10^-7, 9.93366 × 10^-7, 9.92907 × 10^-7, 9.92449 × 10^-7, 9.91991 × 10^-7, 9.91533 × 10^-7, 9.91076 × 10^-7, 9.90618 × 10^-7, 9.90161 × 10^-7, 9.89704 × 10^-7, 9.89247 × 10^-7, 9.88791 × 10^-7, 9.88335 × 10^-7, 9.87878 × 10^-7, 9.87422 × 10^-7, 9.86967 × 10^-7, 9.86511 × 10^-7, 9.86056 × 10^-7, 9.85601 × 10^-7, 9.85146 × 10^-7, 9.84691 × 10^-7, 9.84237 × 10^-7, 9.83782 × 10^-7, 9.83328 × 10^-7, 9.82874 × 10^-7, 9.82421 × 10^-7, 9.81967 × 10^-7, 9.81514 × 10^-7, 9.81061 × 10^-7, 9.80608 × 10^-7, 9.80155 × 10^-7, 9.79703 × 10^-7, 9.7925 × 10^-7, 9.78798 × 10^-7, 9.78347 × 10^-7, 9.77895 × 10^-7, 9.77443 × 10^-7, 9.76992 × 10^-7, 9.76541 × 10^-7, 9.7609 × 10^-7, 9.7564 × 10^-7, 9.75189 × 10^-7, 9.74739 × 10^-7, 9.74289 × 10^-7, 9.73839 × 10^-7, 9.7339 × 10^-7, 9.7294 × 10^-7, 9.72491 × 10^-7, 9.72042 × 10^-7, 9.71593 × 10^-7, 9.71145 × 10^-7, 9.70696 × 10^-7, 9.70248 × 10^-7, 9.698 × 10^-7, 9.69352 × 10^-7, 9.68905 × 10^-7, 9.68457 × 10^-7, 9.6801 × 10^-7, 9.67563 × 10^-7, 9.67116 × 10^-7, 9.6667 × 10^-7, 9.66223 × 10^-7, 9.65777 × 10^-7, 9.65331 × 10^-7, 9.64886 × 10^-7}

D. Figures

Figure 1A: effect of f and ρ on the sign of Δp2 and Δt2 when μ = 0.01, c=0, and s=0.1

{ρ,f}

In the gray region, Δp2 > 0 and Δt2 < 0. In the white region, Δp2 > 0 and Δt2 > 0.

The dashed line indicates the value that f must exceed in order for Δt2 to be positive in the absence of p2 (p2=0).

```
Style["c", Italic]
```

c

```
Figure1A = Show[
  ListPlot[{{{0.001, 0.25}, {0.005, 0.25}, {0.01, 0.25}, {0.1, 0.27},
     {0.2, 0.27}, {0.3, 0.26}, {0.4, 0.25}, {0.5, 0.24},
     {0.6, 0.22}, {0.7, 0.21}}, {{0, 0.38419}, {1, 0.38419}}},
   Joined → True, PlotRange → {{0.0, 0.7}, {0.0, 1.0}},
   Frame → True, FrameTicks → {{True, False}, {True, False}},
   FrameLabel → {"Strength of aversion to T₁ females by P₂ males (ρ)",
     "Fecundity benefit of mating with a T₂ female (f)"},
   ImageSize → Large, BaseStyle → {FontSize → 16},
   PlotStyle → {{GrayLevel[0.6]}, {Black, Thick, Dashed}},
   FrameStyle → Black, Filling → {1 → Axis},
   FillingStyle → GrayLevel[0.6]],
  Graphics[Text["Δp₂ > 0, Δt₂ > 0", {0.35, 0.5}]],
  Graphics[Text["Δp₂ > 0, Δt₂ < 0", {0.35, 0.1}]],
  Graphics[Text["s = 0.1, μ = 0.01, c = 0", {0.55, 0.95}]],
  Graphics[Text["p₂ start = 0.05, t₂ start = 0.8", {0.55, 0.88}]]]
```

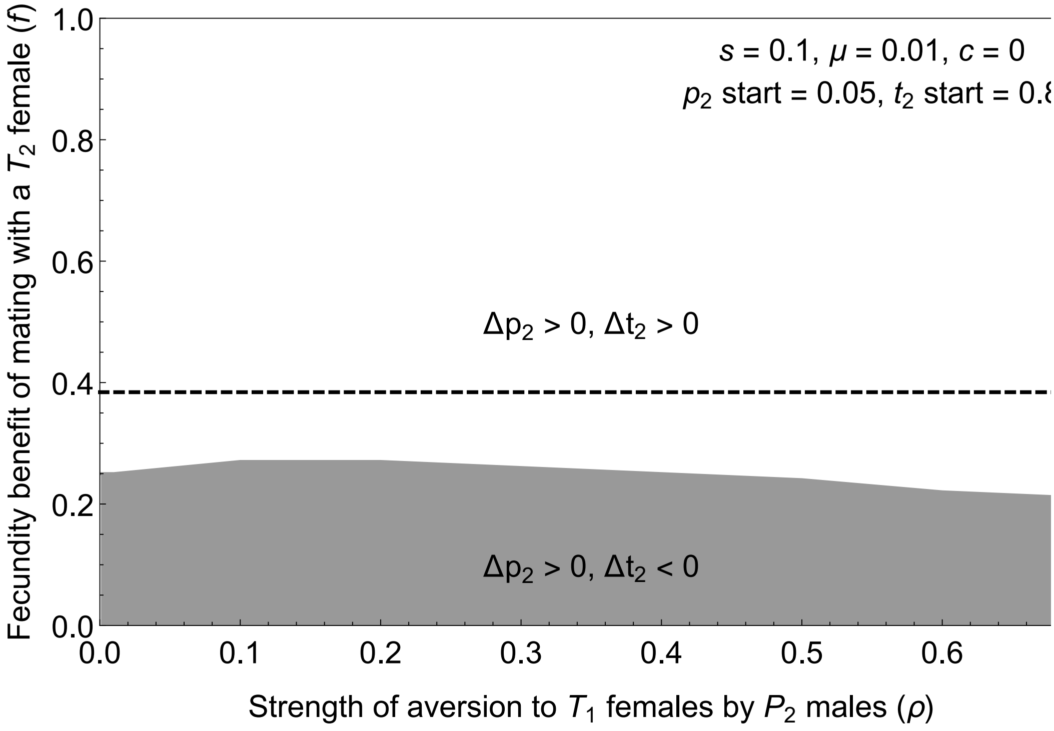
s = 0.1, μ = 0.01, c = 0
p2 start = 0.05, t2 start = 0.8
Δp2 > 0, Δt2 > 0
Δp2 > 0, Δt2 < 0
Fecundity benefit of mating with a T2 female (f)
Strength of aversion to T1 females by P2 males (ρ)
1.0
0.8
0.6
0.4
0.2
0.0
0.0
0.1
0.2
0.3
0.4
0.5
0.6

Figure 1B: effect of f and ρ on the sign of Δp2 and Δt2 when μ = 0.01, c=0, and s=0.2

{ρ,f}

The dashed line indicates the value that f must exceed in order for Δt2 to be positive in the absence of p2 (p2=0).

```
Show[ListPlot[{{{0.001, 0.54}, {0.01, 0.56}, {0.1, 0.6}, {0.2, 0.6},
    {0.3, 0.58}, {0.4, 0.56}, {0.5, 0.52}, {0.6, 0.5}, {0.7, 0.47}},
   {{0.001, 0.14}, {0.01, 0.14}, {0.1, 0.14}, {0.2, 0.14},
    {0.3, 0.14}, {0.4, 0.14}, {0.5, 0.14}, {0.6, 0.14}, {0.7, 0.15}},
   {{0, 0.819438}, {1, 0.819438}}}, Joined → True,
  PlotRange → {{0.0, 0.7}, {0.0, 1.0}}, Frame → True,
  FrameTicks → {{True, False}, {True, False}},
  FrameLabel → {"Strength of aversion to T1 females by P2 males (ρ)",
    "Fecundity benefit of mating with a T2 female (f)"},
  ImageSize → Large, BaseStyle → {FontSize → 16}, PlotStyle →
   {{GrayLevel[0.6]}, {GrayLevel[0.3]}, {Black, Thick, Dashed}},
  FrameStyle → Black, Filling →
   {{1 → {{2}, GrayLevel[0.6]}}, {2 → {Axis, GrayLevel[0.3]}}}],
 Graphics[Text["Δp2 > 0, Δt2 > 0", {0.35, 0.74}]],
 Graphics[Text["Δp2 > 0, Δt2 < 0", {0.35, 0.25}]],
 Graphics[Text["s = 0.2, μ = 0.01, c = 0", {0.55, 0.95}]],
 Graphics[Text["p2 start = 0.05, t2 start = 0.8", {0.55, 0.88}]],
 Graphics[Text[Style["Δp2 < 0, Δt2 < 0", White], {0.35, 0.07}]]]
```

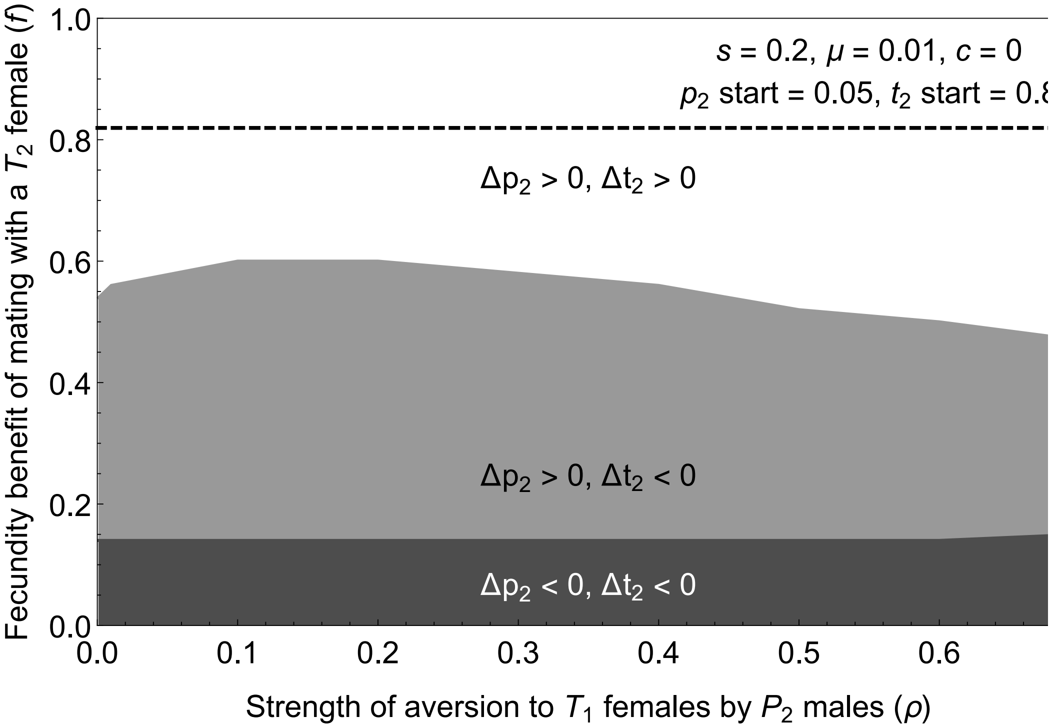

s = 0.2, μ = 0.01, c = 0
p2 start = 0.05, t2 start = 0.8
Δp2 > 0, Δt2 > 0
Δp2 > 0, Δt2 < 0
Δp2 < 0, Δt2 < 0
Fecundity benefit of mating with a T2 female (f)
1.0
0.8
0.6
0.4
0.2
0.0
0.1
0.2
0.3
0.4
0.5
0.6
Strength of aversion to T1 females by P2 males (ρ)

Figure 2A: effect of f and ρ on the sign of Δp2 and Δt2 when μ = 0.01 and c=0.001

{ρ,f}

First line: highest value at which p2 is decreasing and t2 is increasing

Second line: lowest value at which p2 is decreasing and t2 is increasing

```
A2p2dt2i = Show[
  ListPlot[{{{0.001, 0.99}, {0.01, 0.99}, {0.015, 0.99}, {0.02, 0.99}},
    {{0.001, 0.385}, {0.005, 0.385}, {0.012, 0.385}, {0.015, 0.45},
     {0.02, 0.65}}}, Joined → True, PlotRange → {{0.0, 0.7}, {0.0, 1.0}},
   Frame → True, FrameTicks → {{True, False}, {True, False}},
   FrameLabel → {"Strength of aversion to T1 females by P2 males (ρ)",
     "Fecundity benefit of mating with a T2 female (f)"},
   ImageSize → Large, BaseStyle → {FontSize → 16},
   PlotStyle → {GrayLevel[0.5]}, FrameStyle → Black,
   Filling → {{1 → {{2}, GrayLevel[0.5]}}}],
  Graphics[Text["Δp2 < 0,", {0.07, 0.6}]],
  Graphics[Text["Δt2 > 0", {0.065, 0.53}]],
  Graphics[Text["s = 0.1, μ = 0.01, c = 0.001", {0.55, 0.95}]],
  Graphics[Text["p2 start = 0.05, t2 start = 0.8", {0.55, 0.88}]]]
```

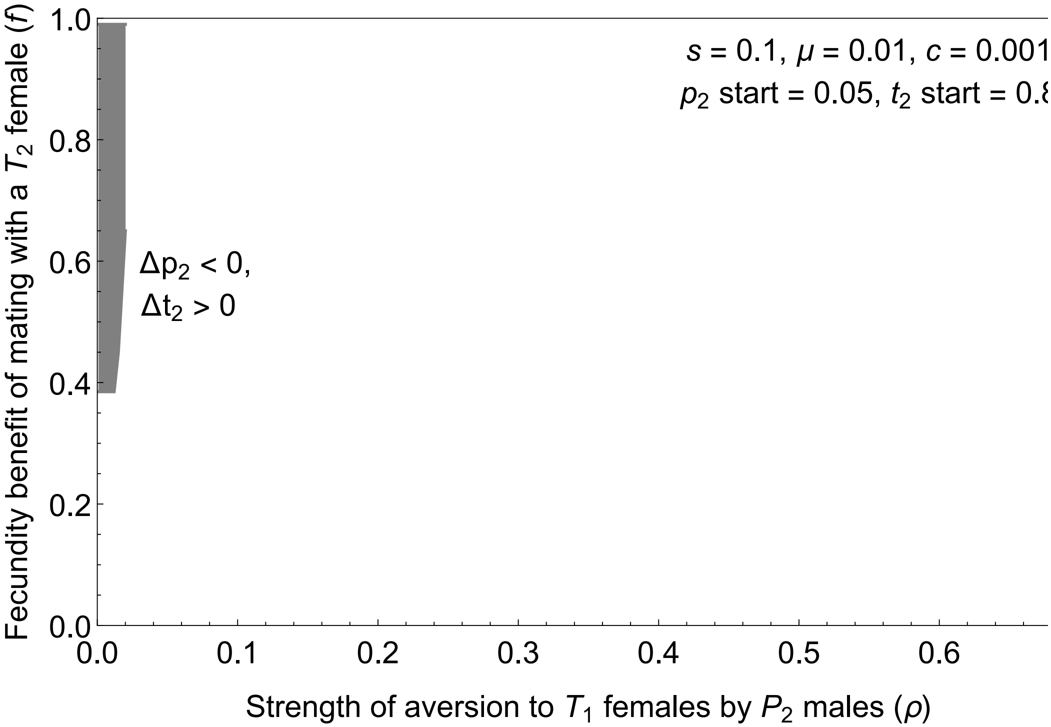
s = 0.1, μ = 0.01, c = 0.001
p2 start = 0.05, t2 start = 0.8
Δp2 < 0,
Δt2 > 0
Fecundity benefit of mating with a T2 female (f)
Strength of aversion to T1 females by P2 males (ρ)
0.0
0.2
0.4
0.6
0.8
1.0
0.0
0.1
0.2
0.3
0.4
0.5
0.6

First line: highest value at which p2 and t2 are both decreasing

Second line: lowest value at which p2 and t2 are both decreasing

```
A2p2dt2d = Show[ListPlot[
   {{{0.001, 0.38}, {0.005, 0.38}, {0.008, 0.155}, {0.015, 0.115},
     {0.025, 0.095}, {0.03, 0.09}, {0.04, 0.085}, {0.05, 0.08},
     {0.1, 0.075}, {0.2, 0.07}, {0.3, 0.07}, {0.4, 0.07},
     {0.5, 0.07}, {0.6, 0.07}, {0.7, 0.075}},
    {{0.001, 0.0}, {0.01, 0.0}, {0.1, 0.0}, {0.2, 0.0},
     {0.3, 0.0}, {0.4, 0.0}, {0.5, 0.0}, {0.6, 0.0}, {0.7, 0.0}},
    {{0, 0.38419}, {1, 0.38419}}}, Joined → True,
   PlotRange → {{0.0, 0.7}, {0.0, 1.0}}, Frame → True,
   FrameTicks → {{True, False}, {True, False}},
   FrameLabel → {"Strength of aversion to T₁ females by P₂ males (ρ)",
     "Fecundity benefit of mating with a T₂ female (f)"},
   ImageSize → Large, BaseStyle → {FontSize → 16}, PlotStyle →
    {GrayLevel[0.3], GrayLevel[0.3], {Black, Thick, Dashed}},
   FrameStyle → Black, Filling → {1 → {{2}, GrayLevel[0.3]}}],
  Graphics[Text[Style["Δp₂ < 0, Δt₂ < 0", White], {0.1, 0.04}]],
  Graphics[Text["s = 0.1, μ = 0.01, c = 0.001", {0.55, 0.95}]],
  Graphics[Text["p₂ start = 0.05, t₂ start = 0.8", {0.55, 0.88}]]]
```

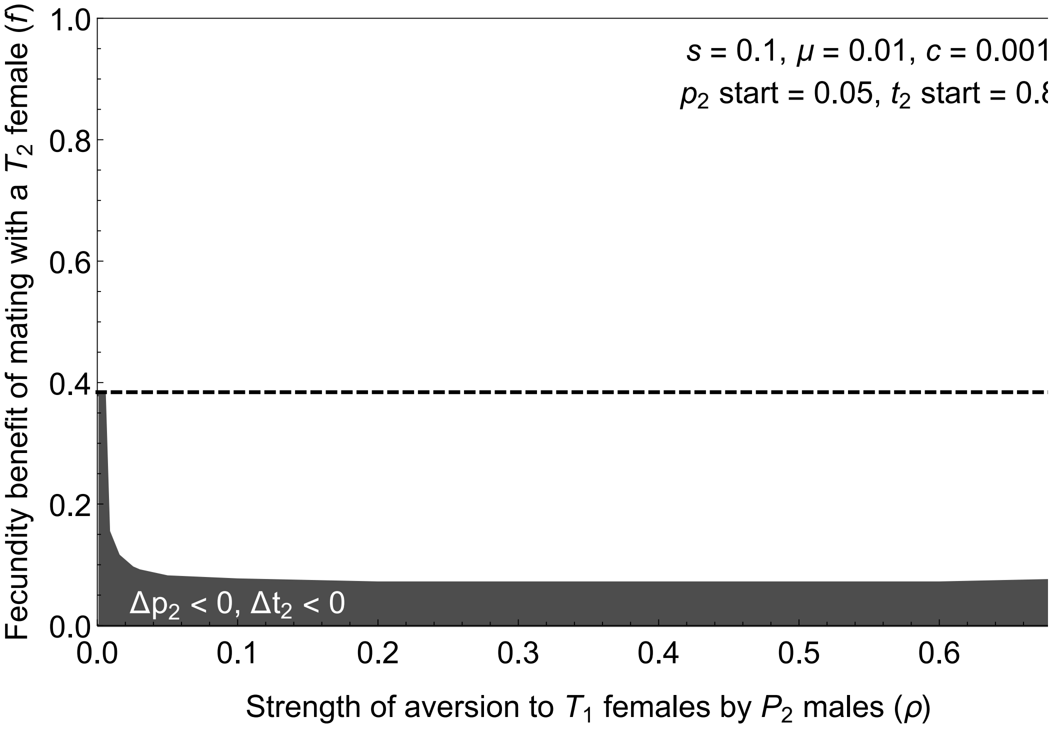

s = 0.1, μ = 0.01, c = 0.001
p2 start = 0.05, t2 start = 0.
Δp2 < 0, Δt2 < 0
Fecundity benefit of mating with a T2 female (f)
1.0
0.8
0.6
0.4
0.2
0.0
0.1
0.2
0.3
0.4
0.5
0.6
Strength of aversion to T1 females by P2 males (ρ)

```
A2p2dt2dextra =
 Show[ListPlot[{{{0.005, 0.38}, {0.008, 0.38}, {0.01, 0.38},
     {0.011, 0.38}, {0.012, 0.38}},
    {{0.005, 0.27}, {0.008, 0.31}, {0.01, 0.34}, {0.012, 0.38}}},
   Joined → True, PlotRange → {{0.0, 0.7}, {0.0, 1.0}},
   Frame → True, FrameTicks → {{True, False}, {True, False}},
   FrameLabel → {"Strength of aversion to T₁ females by P₂ males (ρ)",
     "Fecundity benefit of mating with a T₂ female (f)"},
   ImageSize → Large, BaseStyle → {FontSize → 16}, PlotStyle →
    {GrayLevel[0.3], GrayLevel[0.3], {Black, Thick, Dashed}},
   FrameStyle → Black, Filling → {1 → {{2}, GrayLevel[0.3]}}],
  Graphics[Text[Style["Δp₂ < 0, Δt₂ < 0", White], {0.1, 0.04}]],
  Graphics[Text[Style["s = 0.1, μ = 0.01, c = 0.001", White],
    {0.55, 0.95}]], Graphics[Text[
    Style["p₂ start = 0.05, t₂ start = 0.8", White], {0.55, 0.88}]]]
```

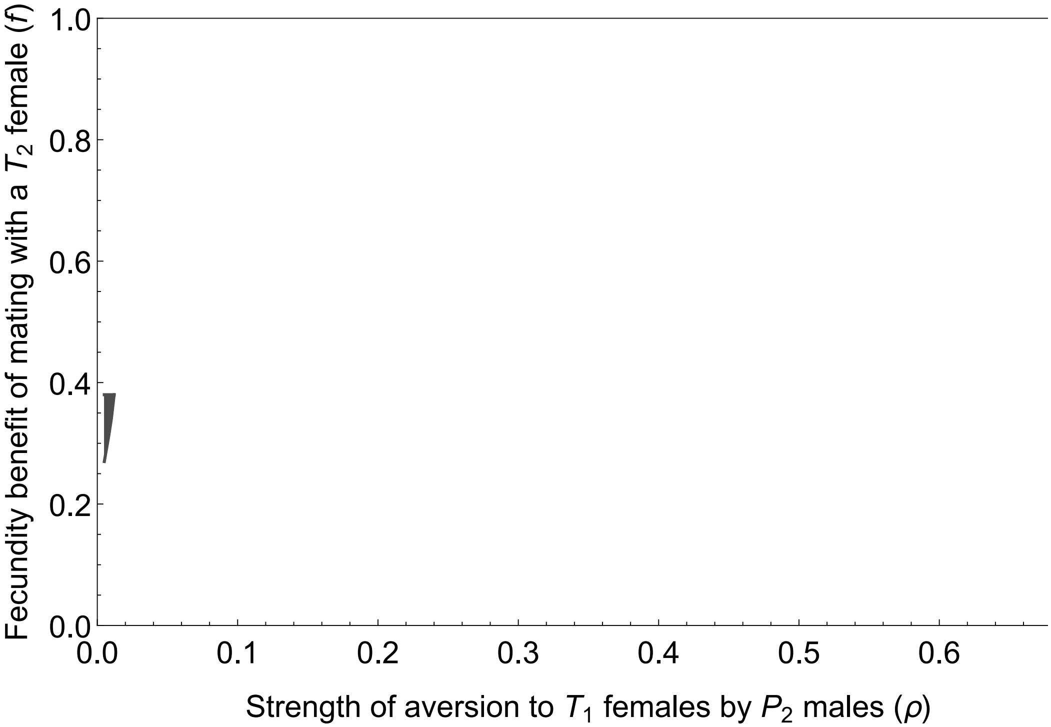
Fecundity benefit of mating with a T2 female (f)
1.0
0.8
0.6
0.4
0.2
0.0
0.0
0.1
0.2
0.3
0.4
0.5
0.6
Strength of aversion to T1 females by P2 males (ρ)

First line: highest value at which p2 is increasing and t2 is decreasing Second line: lowest value at which p2 is increasing and t2 is decreasing

```
A2p2it2d = Show[
  ListPlot[{{{0.005, 0.26}, {0.03, 0.26}, {0.05, 0.265}, {0.1, 0.27},
     {0.2, 0.27}, {0.3, 0.265}, {0.4, 0.25}, {0.5, 0.24}, {0.6, 0.225},
     {0.7, 0.21}}, {{0.005, 0.24}, {0.008, 0.16}, {0.01, 0.145},
     {0.015, 0.12}, {0.025, 0.1}, {0.03, 0.095}, {0.04, 0.09},
     {0.05, 0.085}, {0.1, 0.08}, {0.2, 0.075}, {0.3, 0.075},
     {0.4, 0.075}, {0.5, 0.075}, {0.6, 0.075}, {0.7, 0.08}}},
   Joined → True, PlotRange → {{0.0, 0.7}, {0.0, 1.0}},
   Frame → True, FrameTicks → {{True, False}, {True, False}},
   FrameLabel → {"Strength of aversion to T₁ females by P₂ males (ρ)",
     "Fecundity benefit of mating with a T₂ female (f)"},
   ImageSize → Large, BaseStyle → {FontSize → 16},
   PlotStyle → {GrayLevel[0.8]}, FrameStyle → Black,
   Filling → {{1 → {{2}, GrayLevel[0.8]}}}],
  Graphics[Text["Δp₂ > 0, Δt₂ < 0", {0.5, 0.16}]],
  Graphics[Text["s = 0.1, μ = 0.01, c = 0.001", {0.55, 0.95}]],
  Graphics[Text["p₂ start = 0.05, t₂ start = 0.8", {0.55, 0.88}]]]
```

Printed by Wolfram Mathematica Student Edition

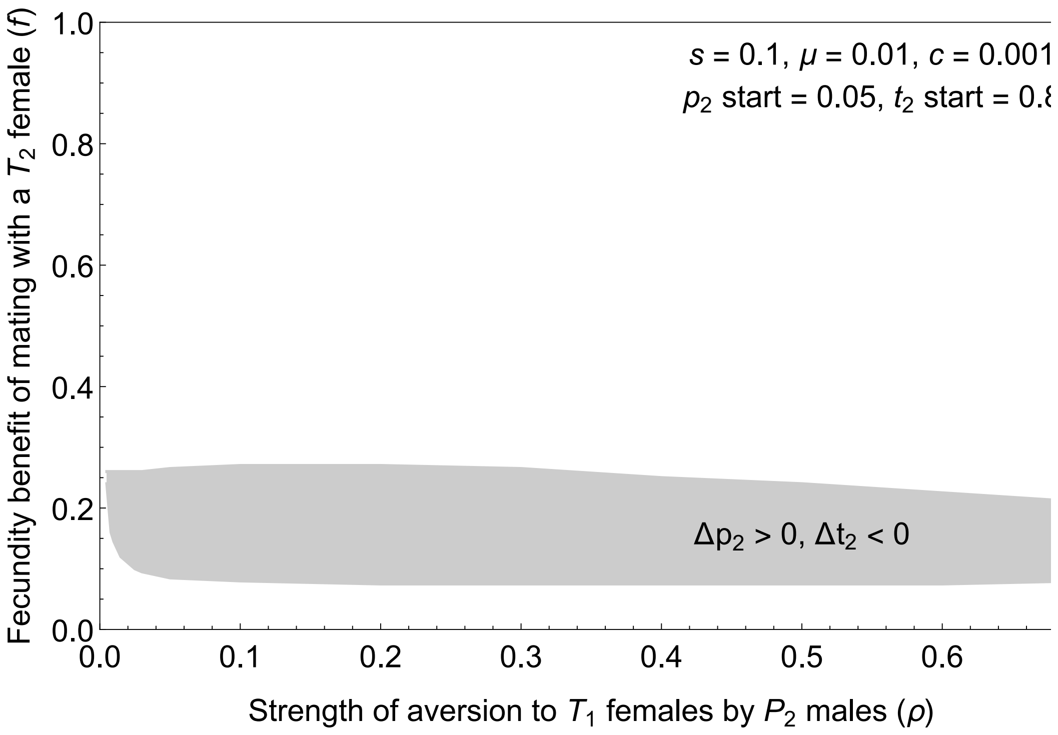
s = 0.1, μ = 0.01, c = 0.001
p2 start = 0.05, t2 start = 0.8
Δp2 > 0, Δt2 < 0
Fecundity benefit of mating with a T2 female (f)
Strength of aversion to T1 females by P2 males (ρ)
1.0
0.8
0.6
0.4
0.2
0.0
0.1
0.3
0.5
0.6

First line: highest value at which p2 and t2 are both increasing

Second line: lowest value at which p2 and t2 are both increasing

```
A2p2it2i =
 Show[ListPlot[{{{0.015, 0.4}, {0.02, 0.6}, {0.025, 0.99}, {0.05, 0.99},
      {0.1, 0.99}, {0.2, 0.99}, {0.3, 0.99}, {0.4, 0.99},
      {0.5, 0.99}, {0.6, 0.99}, {0.7, 0.99}},
     {{0.013, 0.39}, {0.015, 0.39}, {0.02, 0.39}, {0.025, 0.4},
      {0.05, 0.27}, {0.1, 0.275}, {0.2, 0.275}, {0.3, 0.27},
      {0.4, 0.255}, {0.5, 0.245}, {0.6, 0.23}, {0.7, 0.215}}},
    Joined → True, PlotRange → {{0.0, 0.7}, {0.0, 1.0}},
    Frame → True, FrameTicks → {{True, False}, {True, False}},
    FrameLabel → {"Strength of aversion to T₁ females by P₂ males (ρ)",
      "Fecundity benefit of mating with a T₂ female (f)"},
    ImageSize → Large, BaseStyle → {FontSize → 16},
    PlotStyle → {{White}}, FrameStyle → Black,
    Filling → {{1 → {{2}, GrayLevel[1.0]}}}],
  Graphics[Text["Δp₂ > 0, Δt₂ > 0", {0.5, 0.5}]],
  Graphics[Text["s = 0.1, μ = 0.01, c = 0.001", {0.55, 0.95}]],
  Graphics[Text["p₂ start = 0.05, t₂ start = 0.8", {0.55, 0.88}]]]
```

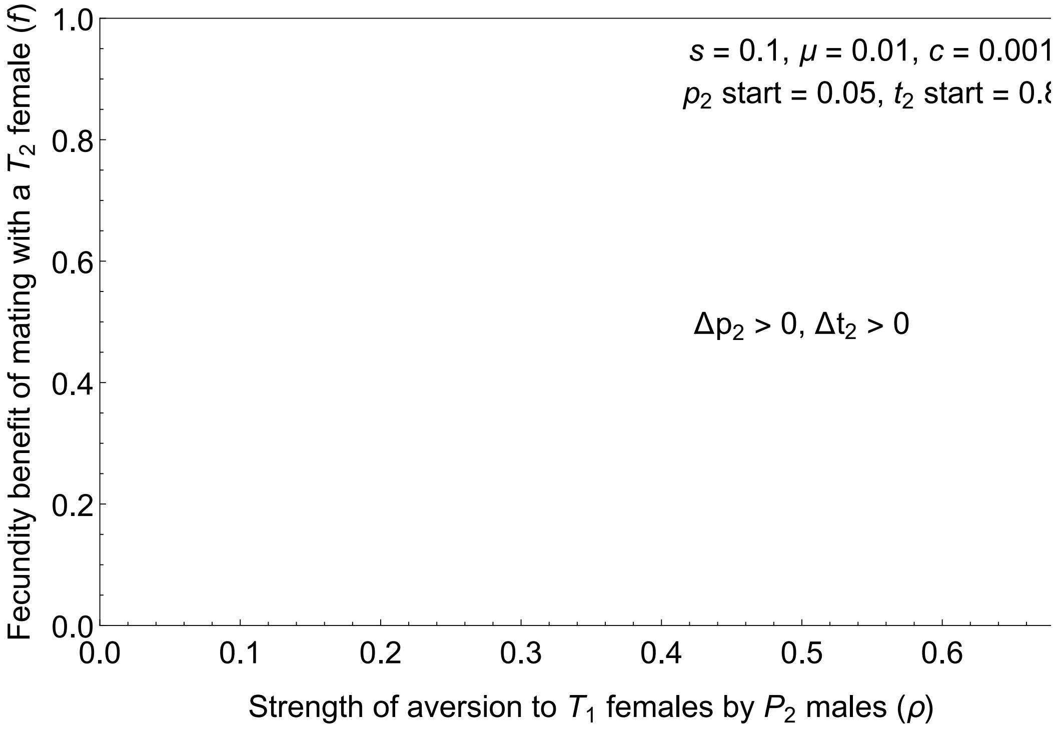
s = 0.1, μ = 0.01, c = 0.001
p2 start = 0.05, t2 start = 0.
Δp2 > 0, Δt2 > 0
Fecundity benefit of mating with a T2 female (f)
1.0
0.8
0.6
0.4
0.2
0.0
0.0
0.1
0.2
0.3
0.4
0.5
0.6
Strength of aversion to T1 females by P2 males (ρ)

```
Figure2A = Show[A2p2it2i, A2p2dt2i, A2p2dt2dextra, A2p2it2d, A2p2dt2d]
```

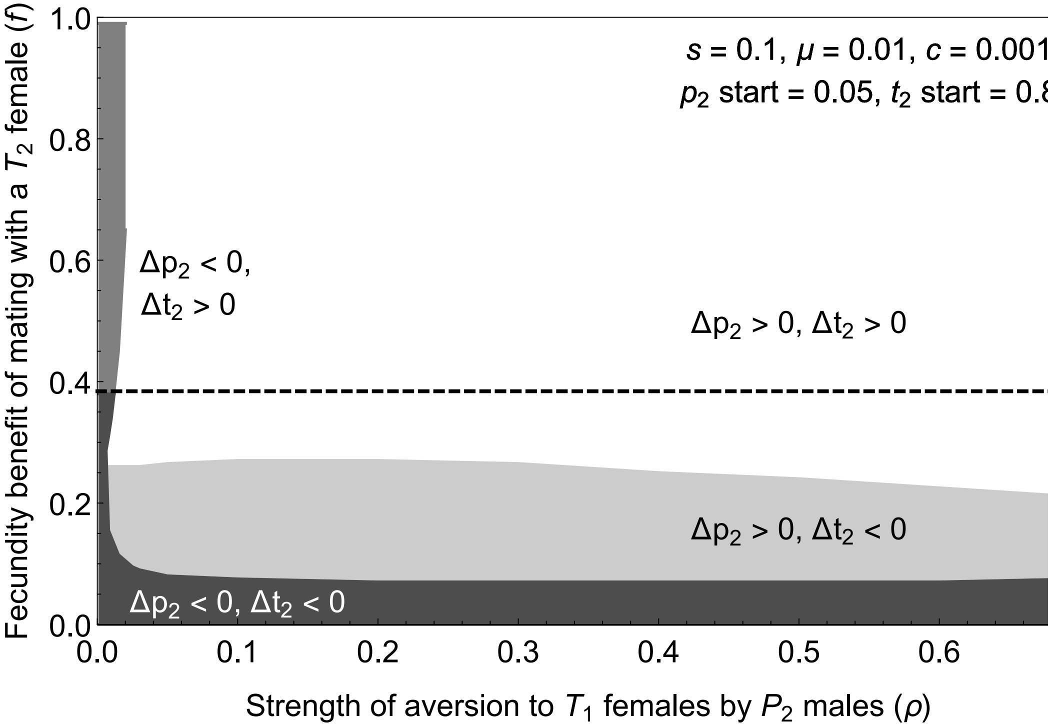

How much do Figures 1A and 2A overlap?

```
Show[Figure2A, Figure1A]
```

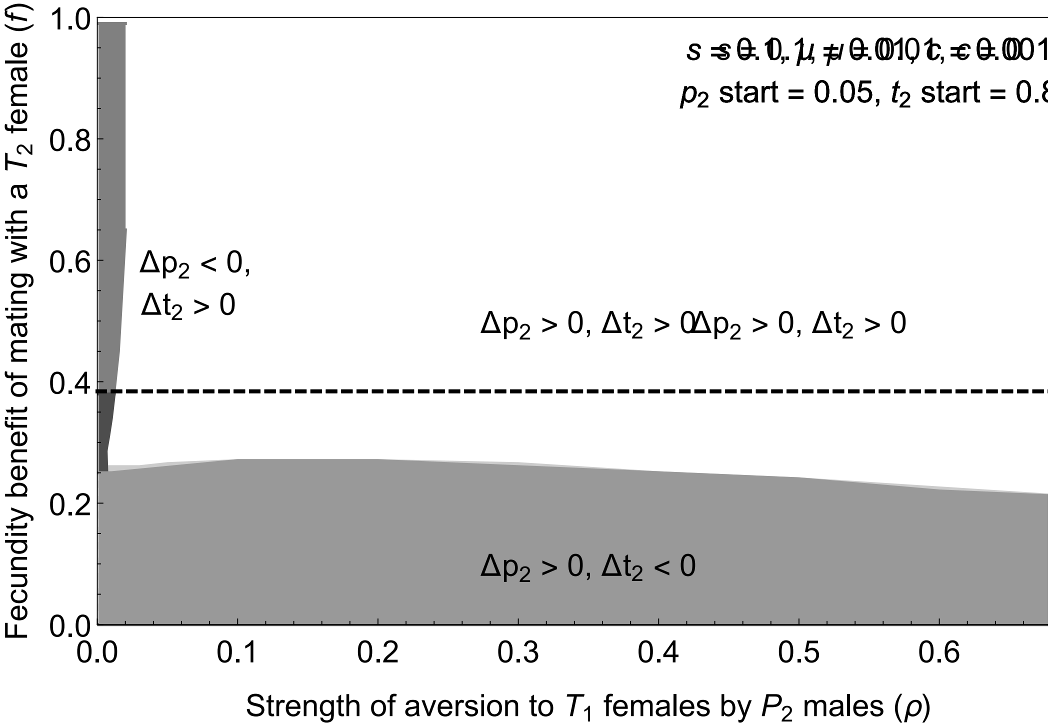

```
Show[Figure1A, Figure2A]
```

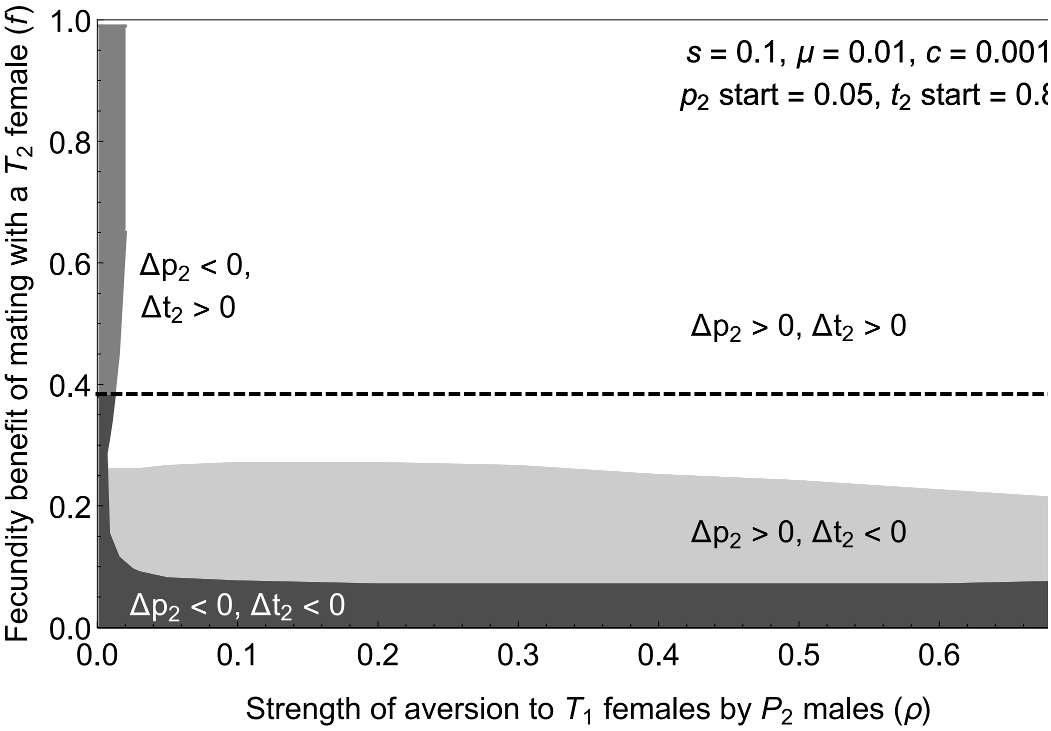

Figure 2B: effect of f and ρ on the sign of Δp2 and Δt2 when μ = 0.01 and c=0.01

{ρ,f}

First line: highest value at which p2 is decreasing and t2 is increasing

Second line: lowest value at which p2 is decreasing and t2 is

increasing

```
B2p2dt2i = Show[ListPlot[{{{0.001, 0.99}, {0.1, 0.99}, {0.24, 0.99}},
    {{0.001, 0.39}, {0.01, 0.275}, {0.025, 0.26}, {0.03, 0.26},
     {0.04, 0.26}, {0.0425, 0.255}, {0.05, 0.27}, {0.1, 0.34},
     {0.15, 0.44}, {0.17, 0.5}, {0.2, 0.65}, {0.24, 0.91}}},
   Joined → True, PlotRange → {{0.0, 0.7}, {0.0, 1.0}},
   Frame → True, FrameTicks → {{True, False}, {True, False}},
   FrameLabel → {"Strength of aversion to T1 females by P2 males (ρ)",
     "Fecundity benefit of mating with a T2 female (f)"},
   ImageSize → Large, BaseStyle → {FontSize → 16},
   PlotStyle → {GrayLevel[0.5]}, FrameStyle → Black,
   Filling → {{1 → {{2}, GrayLevel[0.5]}}}],
  Graphics[Text["Δp2 < 0,", {0.07, 0.6}]],
  Graphics[Text["Δt2 > 0", {0.065, 0.53}]],
  Graphics[Text["s = 0.1, μ = 0.01, c = 0.01", {0.55, 0.95}]],
  Graphics[Text["p2 start = 0.05, t2 start = 0.8", {0.55, 0.88}]]]
```

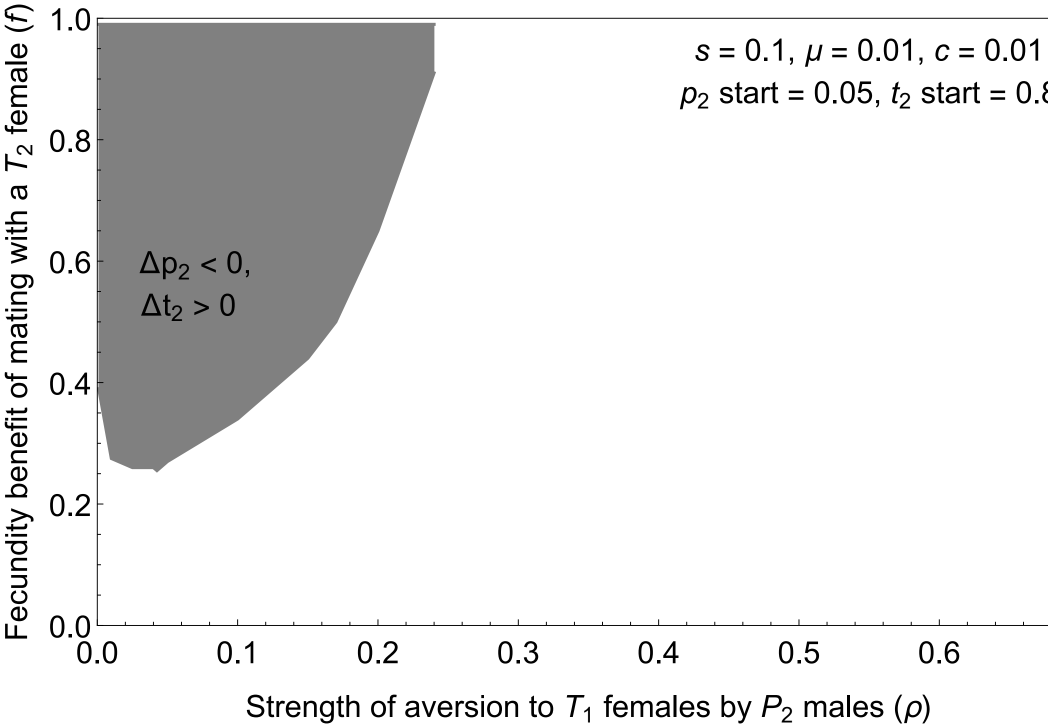
s = 0.1, μ = 0.01, c = 0.01
p2 start = 0.05, t2 start = 0.8
Δp2 < 0,
Δt2 > 0
Fecundity benefit of mating with a T2 female (f)
1.0
0.8
0.6
0.4
0.2
0.0
0.0
0.1
0.2
0.3
0.4
0.5
0.6
Strength of aversion to T1 females by P2 males (ρ)

First line: highest value at which p2 is increasing and t2 is decreasing
Second line: lowest value at which p2 is increasing and t2 is decreasing

```
B2p2it2d = Show[
  ListPlot[{{{0.0425, 0.25}, {0.05, 0.26}, {0.1, 0.26}, {0.15, 0.27},
     {0.2, 0.27}, {0.3, 0.26}, {0.4, 0.25}, {0.5, 0.24},
     {0.6, 0.23}, {0.7, 0.21}}, {{0.0425, 0.235}, {0.05, 0.21},
     {0.1, 0.15}, {0.15, 0.12}, {0.2, 0.11}, {0.3, 0.095},
     {0.4, 0.09}, {0.5, 0.09}, {0.6, 0.09}, {0.7, 0.09}}},
   Joined → True, PlotRange → {{0.0, 0.7}, {0.0, 1.0}},
   Frame → True, FrameTicks → {{True, False}, {True, False}},
   FrameLabel → {"Strength of aversion to T1 females by P2 males (ρ)",
     "Fecundity benefit of mating with a T2 female (f)"},
   ImageSize → Large, BaseStyle → {FontSize → 16},
   PlotStyle → {GrayLevel[0.8]}, FrameStyle → Black,
   Filling → {{1 → {{2}, GrayLevel[0.8]}}}],
  Graphics[Text["Δp2 > 0, Δt2 < 0", {0.5, 0.16}]],
  Graphics[Text["s = 0.1, μ = 0.01, c = 0.01", {0.55, 0.95}]],
  Graphics[Text["p2 start = 0.05, t2 start = 0.8", {0.55, 0.88}]]]
```

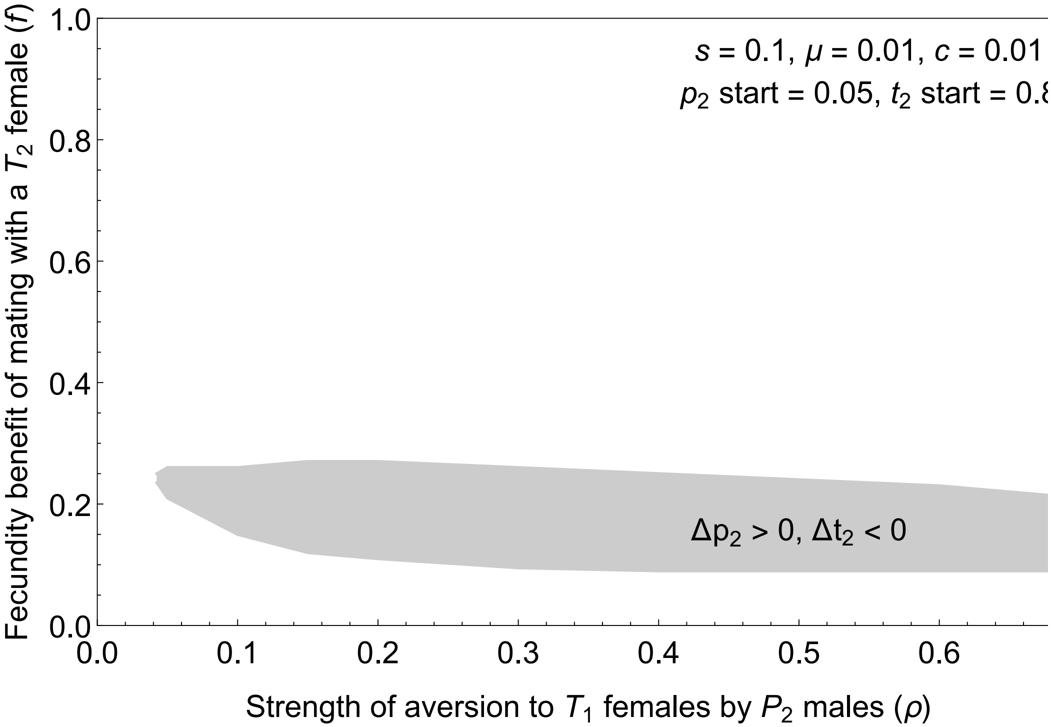

Fecundity benefit of mating with a T2 female (f)
1.0
0.8
0.6
0.4
0.2
0.0
0.0
0.1
0.2
0.3
0.4
0.5
0.6
s = 0.1, μ = 0.01, c = 0.01
p2 start = 0.05, t2 start = 0.8
Δp2 > 0, Δt2 < 0
Strength of aversion to T1 females by P2 males (ρ)

First line: highest value at which p2 and t2 are both increasing

Second line: lowest value at which p2 and t2 are both increasing

```
B2p2it2i =
 Show[ListPlot[{{{0.1, 0.33}, {0.2, 0.6}, {0.3, 0.99}, {0.4, 0.99},
     {0.5, 0.99}, {0.6, 0.99}, {0.7, 0.99}}, {{0.1, 0.27}, {0.2, 0.28},
     {0.3, 0.27}, {0.4, 0.26}, {0.5, 0.25}, {0.6, 0.24}, {0.7, 0.22}}},
   Joined → True, PlotRange → {{0.0, 0.7}, {0.0, 1.0}},
   Frame → True, FrameTicks → {{True, False}, {True, False}},
   FrameLabel → {"Strength of aversion to T₁ females by P₂ males (ρ)",
     "Fecundity benefit of mating with a T₂ female (f)"},
   ImageSize → Large, BaseStyle → {FontSize → 16}, PlotStyle → {{White}},
   FrameStyle → Black, Filling → {{1 → {{2}, GrayLevel[1.0]}}}],
  Graphics[Text["Δp₂ > 0, Δt₂ > 0", {0.5, 0.5}]],
  Graphics[Text["s = 0.1, μ = 0.01, c = 0.01", {0.55, 0.95}]],
  Graphics[Text["p₂ start = 0.05, t₂ start = 0.8", {0.55, 0.88}]]]
```

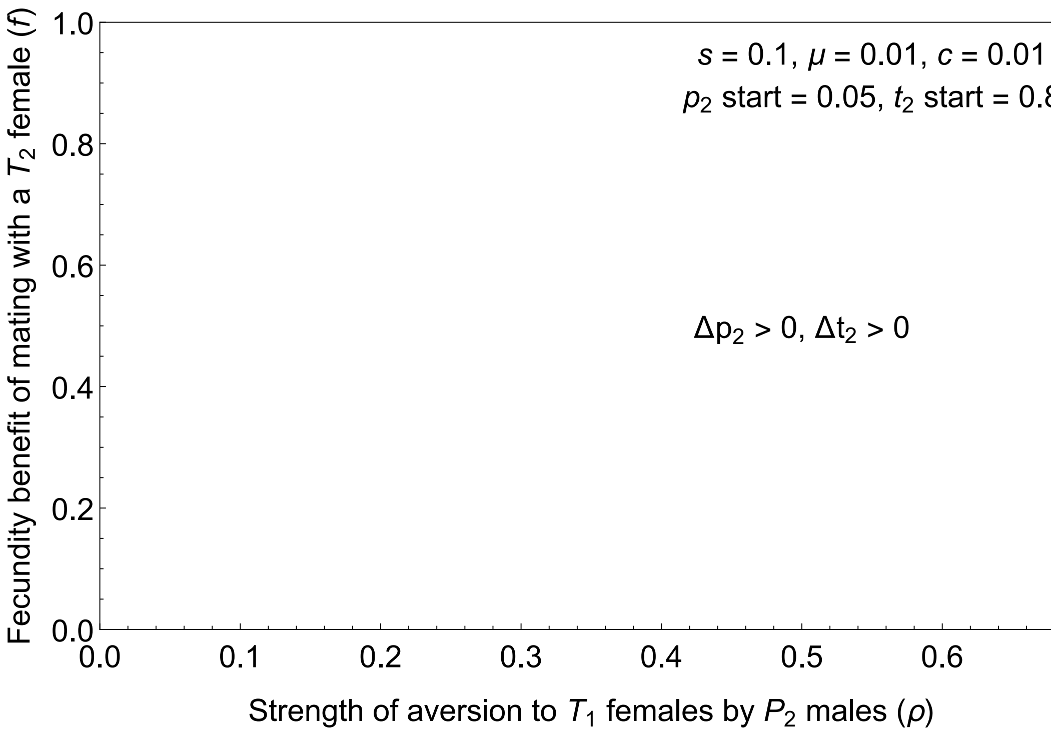

s = 0.1, μ = 0.01, c = 0.01
p2 start = 0.05, t2 start = 0.8
Δp2 > 0, Δt2 > 0
Fecundity benefit of mating with a T2 female (f)
Strength of aversion to T1 females by P2 males (ρ)
1.0
0.8
0.6
0.4
0.2
0.0
0.1
0.2
0.3
0.4
0.5
0.6

First line: highest value at which p2 and t2 are both decreasing

Second line: lowest value at which p2 and t2 are both decreasing

```
B2p2dt2d = Show[
  ListPlot[{{{0.001, 0.38}, {0.01, 0.27}, {0.025, 0.255}, {0.03, 0.255},
     {0.04, 0.255}, {0.0425, 0.23}, {0.05, 0.205},
     {0.1, 0.145}, {0.15, 0.115}, {0.2, 0.105}, {0.3, 0.09},
     {0.4, 0.085}, {0.5, 0.085}, {0.6, 0.085}, {0.7, 0.085}},
    {{0.001, 0}, {0.1, 0}, {0.2, 0}, {0.3, 0}, {0.4, 0}, {0.5, 0},
     {0.6, 0}, {0.7, 0}}, {{0, 0.38419}, {1, 0.38419}}},
   Joined → True, PlotRange → {{0.0, 0.7}, {0.0, 1.0}},
   Frame → True, FrameTicks → {{True, False}, {True, False}},
   FrameLabel → {"Strength of aversion to T₁ females by P₂ males (ρ)",
     "Fecundity benefit of mating with a T₂ female (f)"},
   ImageSize → Large, BaseStyle → {FontSize → 16}, PlotStyle →
    {GrayLevel[0.3], GrayLevel[0.3], {Black, Thick, Dashed}},
   FrameStyle → Black, Filling → {1 → {{2}, GrayLevel[0.3]}}],
  Graphics[Text[Style["Δp₂ < 0, Δt₂ < 0", White], {0.1, 0.05}]],
  Graphics[Text["s = 0.1, μ = 0.01, c = 0.01", {0.55, 0.95}]],
  Graphics[Text["p₂ start = 0.05, t₂ start = 0.8", {0.55, 0.88}]]]
```

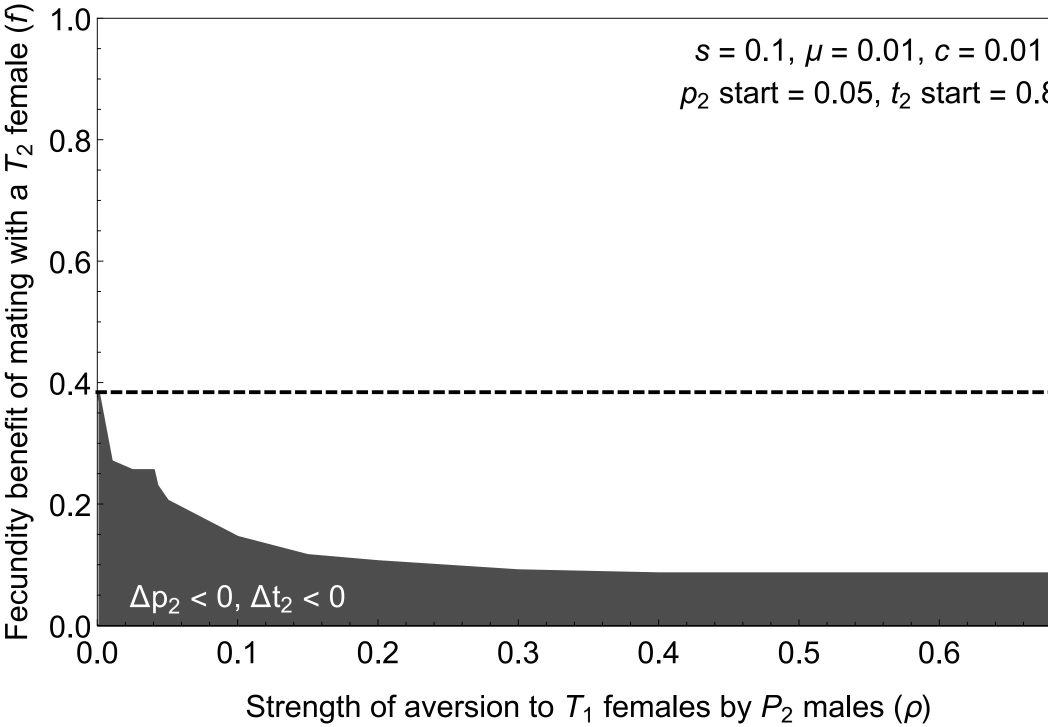
Fecundity benefit of mating with a T_2 female (f)
1.0
0.8
0.6
0.4
0.2
0.0
s = 0.1, μ = 0.01, c = 0.01
p_2 start = 0.05, t_2 start = 0.
$\Delta p_2 < 0$, $\Delta t_2 < 0$
0.0
0.1
0.2
0.3
0.4
0.5
0.6
Strength of aversion to T_1 females by P_2 males (ρ)

```
Figure2B = Show[B2p2dt2i, B2p2it2d, B2p2it2i, B2p2dt2d]
```

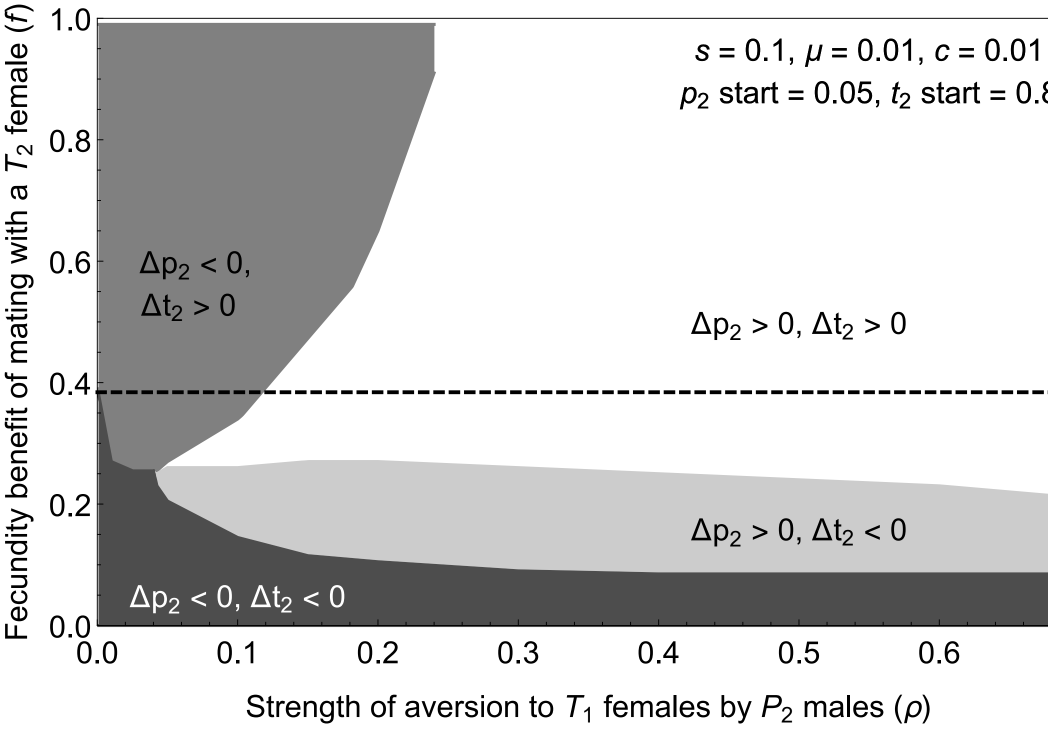

2. Supplementary Figures

Results when the starting frequency of the ornamental trait is low

Figure S1: effect of f and ρ on the sign of Δp2 and

Δt2 when μ = 0.01, c=0, s=0.1, and t2 start = 0.1

{ρ,f}

Compare with figure 1A in the main text.

Fix this: the dashed line indicates the value that f must exceed in order for Δt2 to be positive in the absence of p2 (p2=0).

In[132]:=
```
Show[ListPlot[{{{0.001, 0.24}, {0.1, 0.24}, {0.2, 0.24},
     {0.3, 0.23}, {0.4, 0.22}, {0.5, 0.21}, {0.6, 0.2}, {0.7, 0.19}},
    {{0.001, 0.06}, {0.1, 0.06}, {0.2, 0.06}, {0.3, 0.06},
     {0.4, 0.06}, {0.5, 0.06}, {0.6, 0.07}, {0.7, 0.07}},
    {{0, 0.24994265290988632}, {1, 0.24994265290988632}}},
   Joined → True, PlotRange → {{0.0, 0.7}, {0.0, 1.0}},
   Frame → True, FrameTicks → {{True, False}, {True, False}},
   FrameLabel → {"Strength of aversion to T₁ females by P₂ males (ρ)",
     "Fecundity benefit of mating with a T₂ female (f)"},
   ImageSize → Large, BaseStyle → {FontSize → 16}, PlotStyle →
    {{GrayLevel[0.6]}, {GrayLevel[0.3]}, {Black, Thick, Dashed}},
   FrameStyle → Black, Filling →
    {{1 → {{2}, GrayLevel[0.6]}}, {2 → {Axis, GrayLevel[0.3]}}}],
  Graphics[Text["Δp₂ > 0, Δt₂ > 0", {0.35, 0.44}]],
  Graphics[Text["Δp₂ > 0, Δt₂ < 0", {0.35, 0.15}]],
  Graphics[Text["s = 0.1, μ = 0.01, c = 0", {0.55, 0.95}]],
  Graphics[Text["p₂ start = 0.05, t₂ start = 0.1", {0.55, 0.88}]],
  Graphics[Text[Style["Δp₂ < 0, Δt₂ < 0", White], {0.35, 0.03}]]]
```

Out[132]=

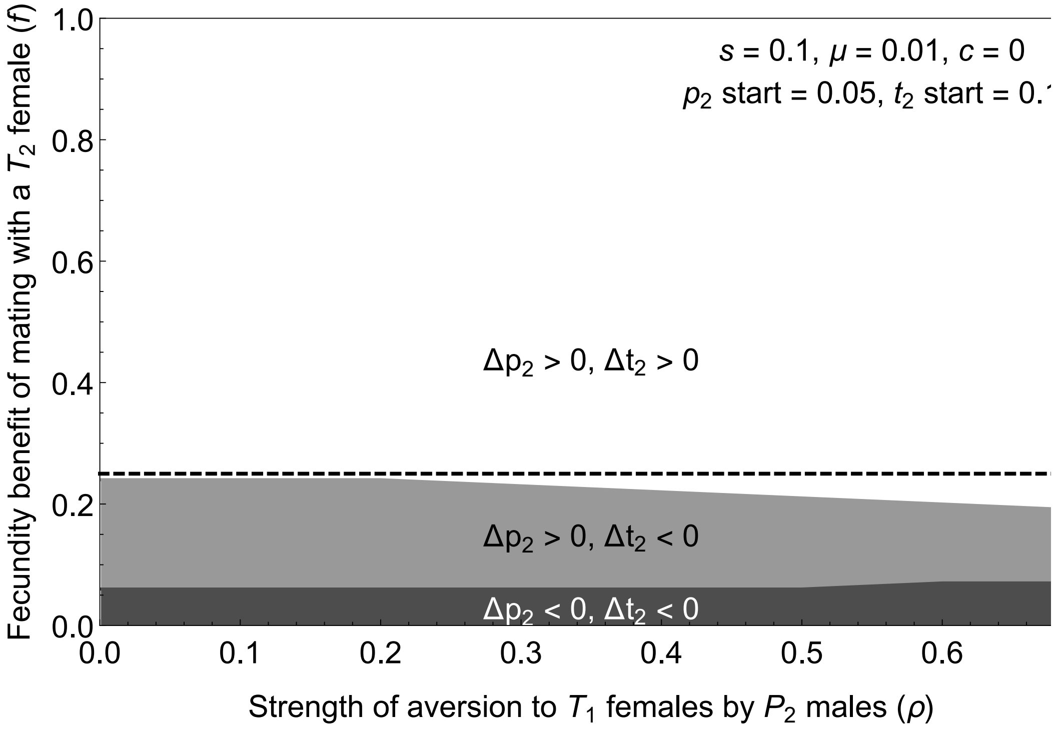
Fecundity benefit of mating with a T2 female (f)
1.0
0.8
0.6
0.4
0.2
0.0
s = 0.1, μ = 0.01, c = 0
p2 start = 0.05, t2 start = 0.
Δp2 > 0, Δt2 > 0
Δp2 > 0, Δt2 < 0
Δp2 < 0, Δt2 < 0
0.0
0.1
0.2
0.3
0.4
0.5
0.6
Strength of aversion to T1 females by P2 males (ρ)

Figure S2: effect of f and ρ on the sign of Δp2 and Δt2 when μ = 0.01, c=0, and s=0.2

{ρ,f}

Compare with figure 1B in the main text.

Fix this: the dashed line indicates the value that f must exceed in order for Δt2 to be positive in the absence of p2 (p2=0).

In[142]:=
```
Show[ListPlot[{{{0.001, 0.53}, {0.1, 0.53}, {0.2, 0.52},
     {0.3, 0.51}, {0.4, 0.49}, {0.5, 0.47}, {0.6, 0.45}, {0.7, 0.43}},
    {{0.001, 0.13}, {0.1, 0.13}, {0.2, 0.13}, {0.3, 0.13},
     {0.4, 0.13}, {0.5, 0.13}, {0.6, 0.13}, {0.7, 0.13}},
    {{0, 0.5395692703536477}, {1, 0.5395692703536477}}},
   Joined → True, PlotRange → {{0.0, 0.7}, {0.0, 1.0}},
   Frame → True, FrameTicks → {{True, False}, {True, False}},
   FrameLabel → {"Strength of aversion to T₁ females by P₂ males (ρ)",
     "Fecundity benefit of mating with a T₂ female (f)"},
   ImageSize → Large, BaseStyle → {FontSize → 16}, PlotStyle →
    {{GrayLevel[0.6]}, {GrayLevel[0.3]}, {Black, Thick, Dashed}},
   FrameStyle → Black, Filling →
    {{1 → {{2}, GrayLevel[0.6]}}, {2 → {Axis, GrayLevel[0.3]}}}],
  Graphics[Text["Δp₂ > 0, Δt₂ > 0", {0.35, 0.64}]],
  Graphics[Text["Δp₂ > 0, Δt₂ < 0", {0.35, 0.25}]],
  Graphics[Text["s = 0.2, μ = 0.01, c = 0", {0.55, 0.95}]],
  Graphics[Text["p₂ start = 0.05, t₂ start = 0.1", {0.55, 0.88}]],
  Graphics[Text[Style["Δp₂ < 0, Δt₂ < 0", White], {0.35, 0.05}]]]
```

Out[142]=

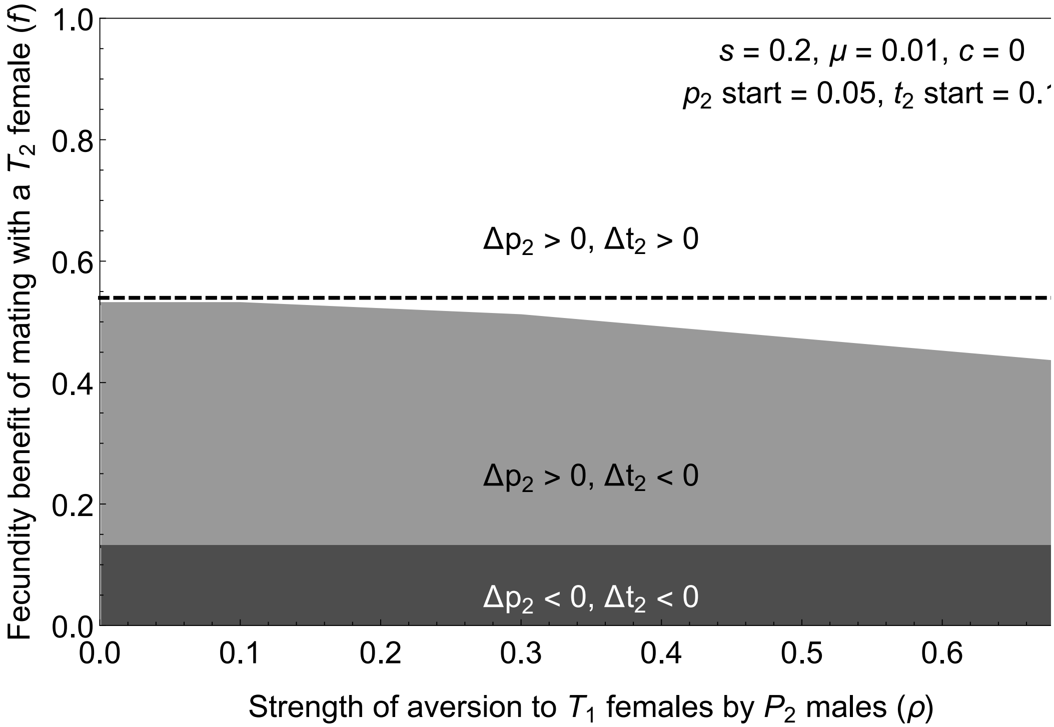

s = 0.2, μ = 0.01, c = 0
p2 start = 0.05, t2 start = 0.
Δp2 > 0, Δt2 > 0
Δp2 > 0, Δt2 < 0
Δp2 < 0, Δt2 < 0
Fecundity benefit of mating with a T2 female (f)
1.0
0.8
0.6
0.4
0.2
0.0
0.1
0.2
0.3
0.4
0.5
0.6
Strength of aversion to T1 females by P2 males (ρ)
